# Yield, cell composition, and function of islets isolated from different ages of neonatal pigs

**DOI:** 10.3389/fendo.2022.1032906

**Published:** 2022-12-21

**Authors:** Hossein Arefanian, Qahir Ramji, Nancy Gupta, Aliya F. Spigelman, Donald Grynoch, Patrick E. MacDonald, Thomas F. Mueller, Lawrence S. Gazda, Ray V. Rajotte, Gina R. Rayat

**Affiliations:** ^1^ Alberta Diabetes Institute, Ray Rajotte Surgical-Medical Research Institute, Department of Surgery, Faculty of Medicine and Dentistry, University of Alberta, Edmonton, AB, Canada; ^2^ Department of Immunology & Microbiology, Dasman Diabetes Institute, Dasman, Kuwait; ^3^ Alberta Diabetes Institute, Department of Pharmacology, Faculty of Medicine and Dentistry, University of Alberta, Edmonton, AB, Canada; ^4^ Alberta Precision Labs, Department of Laboratory Medicine and Pathology, Faculty of Medicine and Dentistry, University of Alberta, Edmonton, AB, Canada; ^5^ Division of Nephrology, University Hospital Zurich, Zurich, Switzerland; ^6^ The Rogosin Institute, Xenia Division, Xenia, OH, United States

**Keywords:** neonatal pig islets, islet transplantation, islet graft rejection, islet graft function, diabetes

## Abstract

The yield, cell composition, and function of islets isolated from various ages of neonatal pigs were characterized using *in vitro* and *in vivo* experimental models. Islets from 7- and 10-day-old pigs showed significantly better function both *in vitro* and *in vivo* compared to islets from 3- and 5-day-old pigs however, the islet yield from 10-day-old pigs were significantly less than those obtained from the other pigs. Since islets from 3-day-old pigs were used in our previous studies and islets from 7-day-old pigs reversed diabetes more efficiently than islets from other groups, we further evaluated the function of these islets post-transplantation. B6 *rag-/-* mouse recipients of various numbers of islets from 7-day-old pigs achieved normoglycemia faster and showed significantly improved response to glucose challenge compared to the recipients of the same numbers of islets from 3-day-old pigs. These results are in line with the findings that islets from 7-day-old pigs showed reduced voltage-dependent K^+^ (Kv) channel activity and their ability to recover from post-hypoxia/reoxygenation stress. Despite more resident immune cells and immunogenic characteristics detected in islets from 7-day-old pigs compared to islets from 3-day-old pigs, the combination of anti-LFA-1 and anti-CD154 monoclonal antibodies are equally effective at preventing the rejection of islets from both age groups of pigs. Collectively, these results suggest that islets from various ages of neonatal pigs vary in yield, cellular composition, and function. Such parameters may be considered when defining the optimal pancreas donor for islet xenotransplantation studies.

## Introduction

Human islet transplantation can be considered a promising option for patients with type 1 diabetes (1). However, its widespread application is currently limited by the shortage of human donors, and using pig islets is a potential solution to this limitation. Neonatal pig islets (NPI) offer an advantage over older pig islets because they are easy to isolate and maintain in culture prior to transplantation (2). They are also more resistant to high glucose- and hypoxia-induced apoptosis (3, 4), have the inherent ability to proliferate and differentiate (2, 5, 6), and are capable of reversing diabetes in both small (2, 7–11) and large animals, including the pre-clinical non-human primate models (12–15). Further, neonatal pigs provide cost benefits due to reduced housing times (16). Thus, these attributes provide a strong rationale to envision the clinical application of NPI xenotransplantation as a therapeutic modality for type 1 diabetes.

For the clinical application of pig islet xenotransplantation, a key component for ensuring control of a validated islet manufacturing process is the use of pre-defined acceptance criteria for the donor pancreas with maximum potential for islet yield (17). Therefore, the age of the donor pigs must be further balanced with the islet yield and functional capacity as these factors can influence the number of required donors with more donors increasing the risk (17, 18). In terms of neonatal pigs, the age of donors which provides a maximum yield of functional islets remains to be elucidated. In this study, we compared the morphological and functional qualities of islets isolated from various ages of neonatal pigs to help define the optimal age of neonatal pigs as islet donors for future clinical islet xenotransplantation.

## Materials and methods

### Animal study

Six- to 8-week-old male immune-competent B6 (C57BL/6J, Jackson Laboratory, Bar Harbor, ME, USA) and immune-deficient B6 *rag-/-* (B6.129S7-Rag1^tm1Mom^/J, Jackson Laboratory) mice were used as recipients of islet transplants. These mice were rendered diabetic by a single intraperitoneal (i.p.) injection of streptozotocin (Sigma, St Louis, MO, USA) at a dose of 180 or 175 mg/kg body weight for B6 or B6 *rag-/-* mice, respectively 4-6 days before transplantation. Blood samples were obtained from the tail vein and glucose levels were monitored using a Precision glucose meter (ONETOUCH, Ultra, Lifescan, Milpitas, CA, USA). Diabetic mice had two consecutive non-fasting blood glucose levels (BGL) ≥20 mmol/l prior to islet transplantation. All mice were cared for according to the guidelines established by the University of Alberta Animal Care and Use Committee and the Canadian Council on Animal Care. Islet donors include 3-, 5-, 7- and 10-day-old male Duroc/Large white cross-neonatal pigs (University of Alberta Swine Research Centre, Edmonton, Alberta, Canada). The use of animals in this study was approved by the University of Alberta Animal Care and Use Committee under protocol number AUP326.

### Islet isolation

NPI were isolated as previously described (2, 12). Briefly, neonatal pigs were anesthetized with isoflurane, followed by subsequent laparotomy and exsanguination. The pancreas was removed by dissection and placed in Hank’s Balanced Salt Solution (HBSS, Sigma) with 0.25% (w/v) bovine serum albumin (BSA, fraction V, Sigma). The pancreas was then chopped with sterile scissors into 1 mm fragments and further digested using Type XI collagenase (1 mg/ml, Sigma). The digested tissue was then poured through a 500 µm nylon mesh filter and cultured for 7 days in Ham’s F10 medium (Sigma), supplemented with 10 mmol/l D-glucose, 50 µmol/l isobutylmethylxanthine (ICN, Biomedicals, Montreal, QC, Canada), 0.5% BSA, 2 mmol/l L-glutamine, 3 mmol/l CaCl_2_, 10 mmol/l nicotinamide (BDH Biochemical, Poole, England), 100 U/ml penicillin, and 100 µg/ml streptomycin at 37°C, 5% CO_2_, and 95% air. The media of isolated islets was changed on days 1, 3, 5, and 7 post-isolations. On day 7, the islets were collected, and aliquots were counted in islet equivalent (I.E.), with 150 µm representing the standard for one I.E. (19–21).

The adult porcine islets were prepared from Newsham sows (Rogosin Institute, Xenia, Ohio, USA) that were over two years of age and had a history of multiple parities. Islets were isolated as previously described (22), with the following enzyme modifications: retrieved pancreases were perfused with 1.7 ml/g pancreas weight of Cold Storage Purification Stock Solution (CSPSS; Mediatech, Inc., Manassas, VA, USA) containing Liberase MTF/Thermolysin at 7.5 U/g pancreas, (Roche, Indianapolis, IN, USA) and 2.5 mg/pancreas Pulmozyme (Genentech, South San Francisco, CA, USA). The distended pancreas was digested at 31°C for approximately 45 minutes and then washed through a mesh basket made of 533 µm stainless steel screen using approximately 15 l of cold HBSS with 2% porcine serum. Digested tissue was centrifuged and resuspended in 1 ml packed tissue per 10 ml of 1.105 g/cm^2^ Euro-Collins Ficoll. Islets were purified on discontinuous Euro-Collins Ficoll gradients and resuspended in RPMI with 10% porcine serum.

### Islet transplantation

500, 1000, or 2000 I.E. from various ages of pigs were transplanted under the left kidney capsule of diabetic B6 *rag-/-* or B6 mice as previously described (2, 9, 10). Islet engraftment was considered successful when the blood glucose levels of recipients reached <10 mmol/l post-transplantation. Rejection of islet xenograft in B6 mice was defined as the first of three consecutive days of hyperglycemia (>10 mmol/l) and was confirmed by histological analysis of the graft.

### Oral glucose tolerance test (OGTT)

At >130 days post-transplantation, an OGTT was performed in randomly selected B6 *rag-/-* mouse recipients of NPI and in naïve non-transplanted *B6 rag-/-* mice (control group) following our previously described method (2, 9). Briefly, naïve non-transplanted *B6 rag-/-* mice and islet-transplant recipient *B6 rag-/-* mice that had maintained stable normal blood glucose levels (<10.0 mmol/l) were fasted for approximately 16 hours, and provided with an unlimited supply of drinking water. A glucose tolerance test was performed on these mice by administration of 50% dextrose solution (2.0 g/kg body weight) by oral gavage. Blood samples were obtained from the tail vein and glucose levels were measured at 0, 15, 30, 60, and 120 minutes after glucose administration using a Precision glucose meter (ONETOUCH, Ultra, Lifescan). The mice were returned to the housing facility and their blood glucose levels were measured once per week. At 150 days post-transplantation, the kidney that contained the islet xenograft was procured and blood glucose was measured until the mice returned to the diabetic state.

### Monoclonal antibody therapies

B6 mouse recipients of islets from 3- and 7-day-old neonatal pigs were randomly designated to receive short-term i.p. injections of anti-LFA-1 mAb (KBA; rat IgG2a) at 200 µg on days 0, 1, 7, 14 post-transplant plus anti-CD154 mAb (MR-1; hamster IgG1; Bio Express, West Lebanon, NH, USA) at 250 µg on days –1 and 1 and 2 times a week for an additional 4 weeks post-transplant based on our published protocol (7, 9).

### DNA and insulin content measurements

Five hundred µl of NPI were washed and diluted with HBSS (Sigma) medium to a final volume of 10 ml. Then, duplicate aliquots of 200 µl were taken from the diluted sample of islets and homogenized by ultrasonication on ice in distilled water. A sample of the homogenate was analyzed in duplicate for DNA content using a fluorometric method based on diaminobenzoic acid-induced fluorescence (23). The remaining homogenate was extracted overnight in 70% acid ethanol (0.3 N HCl in absolute ethanol). Insulin was measured from a 1:20 dilution of the extract using a commercial radioimmunoassay kit cross-reacting with porcine and human insulin (Linco, St Charles, MO, USA). Values are presented as µg of DNA or insulin content in 1 ml of NPI or approximately 2,000 I.E.

### Static glucose stimulation assay

To assess the insulin secretory ability of NPI, a static glucose stimulation assay was performed at 37°C, 5% CO_2_, and 95% air. 100 I.E. from various ages of pigs were washed with HBSS medium, incubated for 120 minutes in Ham’s F10 medium (Sigma) supplemented with 2mmol/l L-glutamine, 0.5% (w/v) BSA, and either low (2.8 mmol/l) or high (20.0 mmol/l) glucose at 37°C, 5% CO_2_, and 95% air. Islets and culture medium were then separated by low-speed centrifugation and assayed for their respective insulin content using the method described above. Stimulation indices (SI) were calculated by dividing the amount of insulin released after stimulation with high glucose by that released after stimulation with low glucose.

### Immunostaining

Islets were dissociated into single cells, placed on slides, fixed in 10% (v/v) buffered formalin solution, and kept in 70% (v/v) ethanol following our published protocol (24). Cells were incubated with primary antibodies for 30 minutes, followed by washing and incubating with appropriate biotinylated secondary antibodies for 20 minutes (**Supplementary Material**). Avidin-biotin complex/horseradish peroxidase (Vector Laboratories) and 3, 3-diaminobenzidine tetrahydrochloride (BioGenex, San Ramon, CA, USA) were used to detect positive-stained cells (brown color). 500 cells were counted by two individuals using a light microscope and the mean percentages of positive-stained cells were calculated. The presence of insulin-positive cells in grafts was also examined following our published protocol (7–10). Briefly, the kidney that bears the islet graft was fixed in 10% buffered formalin solution and embedded in paraffin, then 5 µm sections were stained with guinea pig anti-insulin primary antibody (1:1000; DAKO laboratories, Mississauga, ON, Canada) for 30 min, followed by the addition of biotinylated goat anti-guinea pig IgG secondary antibody (1:200; Vector Laboratories). Avidin-biotin complex/horseradish peroxidase (ABC/HP; Vector Laboratories, Burlingame, CA, USA) and 3, 3-diaminobenzidinetetrahydrochloride (DAB; BioGenex, San Ramon, CA, USA) were used to detect positive cells (brown color). All paraffin-embedded tissue sections were counter-stained with Harris’ hematoxylin and eosin.

### Kv channel and voltage-dependent K^+^ and Ca^2+^ channel activity measurements

The Kv channel activity was measured using the Kv intracellular and extracellular solutions and the Voltage-dependent Ca^2+^ channels (VDCCs) activity was measured using VDCC intracellular and extracellular solutions (**Supplementary Material**). Patch pipettes, pulled from thin-walled borosilicate glass and coated with Sylgard, had resistances of 4–6 megaohm (MΩ) when filled with the pipette solution. The currents were normalized to cell size and were expressed as picoampere per picofarad (pA/pF). All experiments were performed using the standard whole-cell technique with an EPC10 amplifier and Patchmaster software (HEKA Electronics, Lambrecht/Pfalz, Germany) at room temperature. Cells were positively identified as beta cells following the protocol for insulin immunostaining as previously described.

### Flow cytometry

Islets were dissociated into single cells by gentle agitation in calcium-free medium containing 15 μg/ml trypsin (Boehringer Mannheim, Laval, QC, Canada) and 4 μg/ml DNase (Boehringer Mannheim) following our published protocol (24). The cell suspension was filtered through a 63-μm nylon screen to remove cell clumps and then centrifuged through Percoll (Sigma) of 1.040 g/ml density to eliminate dead cells and debris. Islet cells were counted using trypan blue exclusion dye and a hemocytometer. Adult porcine peripheral blood mononuclear cells (PBMCs) were used as controls and were isolated following our standard protocol. Briefly, peripheral blood was diluted in a 1:1 ratio with saline. Forty ml of diluted blood were overlaid on 10 ml of lympholyte mammal (Cedarlane Inc., Hornby, ON, Canada) and spun for 20 minutes at 1500 rpm. The PBMCs were collected, washed with saline, and counted using trypan blue and a hemocytometer. Aliquots of 0.5 x 10^6^ live islet cells and adult porcine PBMCs were incubated for 30 minutes at 4°C with 1:100 dilution of antibodies specific for immune cell markers (**Supplementary Figure 1**). Fluorescence histograms were created using BD FACS Calibur flow cytometry machine (BD Biosciences, Mississauga, ON, Canada), with a gate on live cells, and were used to determine the percentage of positive cells labeled with the corresponding antibodies (**Supplementary Figure 1**). Analysis of the immune cell population was performed using BD FACSDivaTM software (BD Biosciences).

### Microarray analysis

Islets isolated from 3- and 7-day-old pigs (n=4) were processed for analysis with pig Affymetrix^®^ GeneChip microarrays (No.: 900623) representing 24,123 probe sets (of which 5,301 were annotated corresponding to 1,859 unique genes). The total RNA was isolated using the RNeasy Mini Kit (QIAGEN, Valencia, CA, USA), and amplified according to the Affymetrix^®^ protocol (Santa Clara, CA, USA). If the starting input of cRNA was below 2.5 µg an additional round of linear amplification was conducted. RNA yields were measured by UV absorbance and RNA fixed and acute features assessed by Agilent Bioanalyzer. RNA labelling and hybridization of the Affymetrix^®^ GeneChip microarrays of each sample were carried out according to the Affymetrix^®^ GeneChip Expression Analysis Manual (Thermo Fisher Scientific, Waltham, MA, USA).

Raw microarray data were normalized to the median of each probe set across all samples. Afterward, high throughput computer software (GeneSpring GX, version 11.5, Agilent Technologies, Santa Clara, CA, USA) was used to determine differentially expressed probe sets and genes between islets from 3- and 7-day-old pigs. A principal component analysis of the samples was performed for qualitative analysis as well as to identify outliers and the overall differentiation between the samples. In a filtering step, probe sets that are lowly expressed across all samples (<20%) were eliminated, leaving 20,056 probe sets for statistical analysis. An unpaired t-test (corrected p-value <0.05, Multiple Testing Correction by Benjamini-Hochberg) was used to determine differentially expressed probe sets between islets from 3- and 7-day-old pigs. Moreover, a fold-change analysis was used to select genes with >1.5- and 2-fold changes (**Supplementary Tables 1**, **2**). A Gene Ontology (GO) analysis was performed on the up-regulated genes with a fold-change >2 in islets from 7-day-old compared to 3-day-old pigs (adjusted p-value <0.1). To investigate pathways, significant genes with a fold-change >1.5 were imported into Ingenuity Pathway Analysis (IPA, www.ingenuity.com) and run through a core analysis. In IPA, canonical pathways were identified and ranked according to p-value (Fisher’s Exact Test p-value).

### Induction of hypoxia and reoxygenation stress

After 7 days of culture, 500 I.E. from 3- or 7-day-old pigs were placed in a 40 μm Corning cell strainer per well of a six-well tissue culture plate. Hypoxia stress was induced using a hypoxia chamber (Stemcell Technologies, Vancouver, Canada), which was purged with a gas mixture of 1% O_2_, 5% CO_2_, and 94% N_2_ for 15 minutes (10 l/minute), and then the sealed chamber was placed inside a tissue culture incubator maintained at 37°C (25). After 24 hours, the plates were removed and either analyzed immediately or further cultured for 24 hrs under normal conditions (37°C, 5% CO_2_, 95% air) to induce reoxygenation stress. Results were compared to control islets cultured under hypoxic and normal conditions.

### Statistical analysis

Data are expressed as mean ± SEM of n independent observations. Statistical significance of differences in insulin, DNA, pancreatic hormones, and porcine antigens between groups was calculated using Kruskal Wallis one-way analysis of variance and two-tailed unpaired Student’s t-tests. All statistical analysis was performed using GraphPad Prism 6 software or SPSS version 25 (SPSS Inc., Chicago, IL, USA). Analysis of Kv and calcium channels between groups was performed using FitMaster software (HEKA Electronik, Reutlingen, Germany). P <0.05 was considered statistically significant.

## Results

### Morphological characteristics of islets from various ages of neonatal pigs

**Figure 1:**
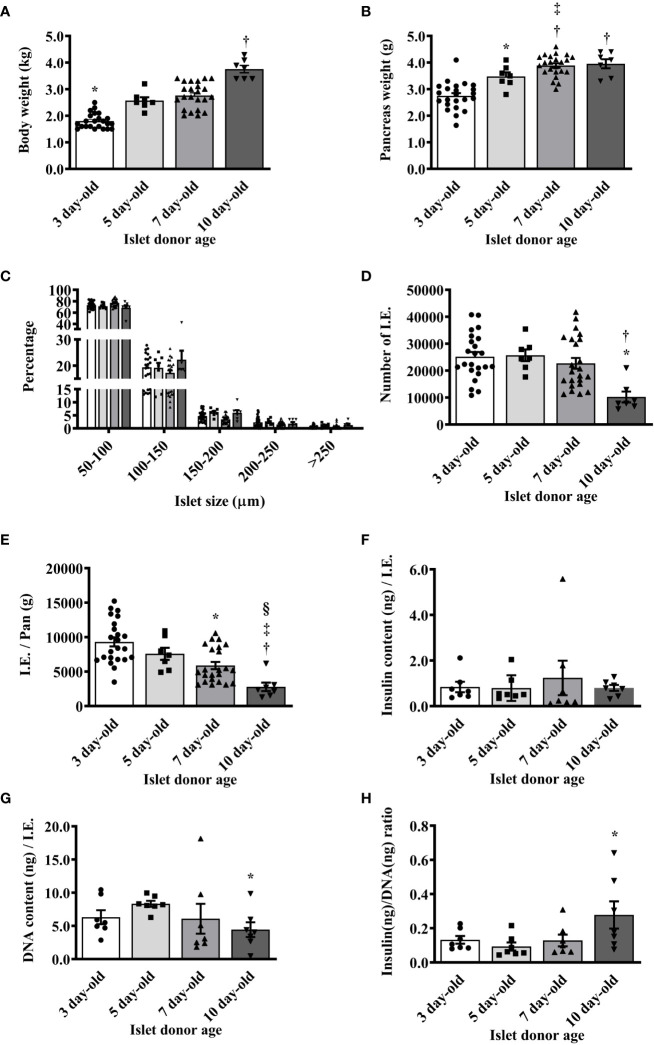
Morphological characteristics of islets isolated from neonatal pigs of various ages. **(A)** Mean body weight of neonatal pigs (*p <0.0001, 3-day-old vs. 5-, 7-, and 10-day-old; †p <0.0001, 5- and 7-day-old vs. 10-day-old). **(B)** Mean pancreas weight of neonatal pigs (*p <0.005 between 3- and 5-day-old; †p <0.0001, 3-day-old vs. 7- and 10-day-old; ‡p <0.05 between 5- and 7-day-old). **(C)** Proportions of islets isolated from various ages of neonatal pigs based on size. **(D)** Islet yield expressed in I.E. (*p <0.0005, 3- and 5-day-old vs. 10-day-old; †p <0.005 between 7- and 10-day-old). **(E)** Islet yield per pancreas weight of neonatal pigs (*p <0.0005 between 3- and 7-day-old; †p <0.0001 between 3- and 10-day-old; ‡p <0.001 between 5- and 10-day-old; §p ≤0.005 between 7- and 10-day-old). Data from 3- (n=23), 5- (n=7), 7- (n=23), and 10- (n=7) day-old pigs are shown. **(F)** Insulin content per I.E. from various ages of neonatal pigs. **(G)** DNA content per I.E. from various ages of neonatal pigs (*p <0.01 between 5-day-old vs. 10-day-old). **(H)** Insulin to DNA content ratio detected in islets from various ages of neonatal pigs (*p <0.05 between 5- and 10-day-old). Data from 3-, 5-, 7-, and 10-day-old pigs (n=7 for each group) are shown.

The weights of neonatal pigs and their pancreas were initially measured prior to islet isolation. As expected, the average body and pancreas weights significantly increased as the age of pigs increased (**Figures 1A, B**). However, we found no significant difference in the islet size distribution between the islet preparations from different ages of pigs (**Figure 1C**). In these preparations, there were more 50 to 100 µm islets followed by 100 to 150 µm islets. The average islet yields were comparable between 3-, 5-, and 7-day-old pigs but significantly lower in 10-day-old pigs (**Figure 1D**). As such, the I.E. per weight of the pancreas of different ages of pigs were significantly decreased as their age increased (**Figure 1E**). There was no significant difference in the total insulin content/I.E. observed between the different ages of pigs (**Figure 1F**). The total DNA content however significantly varies between the groups. Higher amounts of DNA/I.E. were observed in islets from 5-day-old followed by islets from 3-, 7-day-old and lowest in islets from 10-day-old pigs (**Figure 1G**). Collectively, these results accounted for a relatively higher insulin/DNA content ratio detected in islets from 10-day-old pigs compared to the other groups (**Figure 1H**).

Characterization of the endocrine cells in islets from 7-day-old pigs shows a lower proportion of insulin-positive cells compared to the proportion of these cells in islets from other ages of pigs. The percentage of insulin-positive cells was highest in 10-day-old pigs compared to the other groups (**Table 1**). However, these results were not found to be statistically significant between the groups. Islets from 3-day-old pigs display more glucagon, somatostatin, and pancreatic polypeptide-positive cells compared to islets from other ages of pigs. Islets from 10-day-old pigs showed a significantly lower proportion of CK7-positive precursor endocrine cells compared to islets from younger pigs (**Table 1**).

**Table 1:** Cell composition of islets from various ages of neonatal pigs.

Mean Percentage of Stained Positive Cells ± SEM						
Group	n	Insulin	Glucagon	Somatostatin	Pancreatic Polypeptide	CK7
**3-day-old**	5	28.61 ± 1.62	28.02 ± 1.57	6.47 ± 0.80	6.12 ± 0.62	15.37 ± 2.58^#^
**5-day-old**	5	27.31 ± 3.06	19.40 ± 3.16*	4.01 ± 0.58^‡^	3.40 ± 0.23^§^	15.07 ± 4.37
**7-day-old**	4	24.22 ± 1.93	19.37 ± 1.48^†^	3.26 ± 1.33	2.68 ± 0.90^‖^	16.86 ± 2.38**
**10-day-old**	4	35.19 ± 6.59	23.36 ± 3.47	4.51 ± 0.57	3.84 ± 0.41_¶_	2.59 ± 2.21

Islets were dissociated into single cells, placed on slides, fixed in 10% (v/v) buffered formalin solution, and kept in 70% (v/v) ethanol following our published protocol. Islet cells were incubated with antibodies to endocrine hormones. Two individuals counted positive-stained cells out of 500 islet cells from each preparation. The mean counts were calculated for each sample.

*p <0.05 between 3- and 5- day-old.

^†^p <0.01 between 3- and 7-day-old.

^‡^p <0.05 between 3- and 5-day-old.

^§^ p<0.005 between 3- and 5-day-old.

^‖^p ≤0.01 between 3- and 7-day-old.

^¶^p <0.05 between 3-and 10-day-old.

^#^ p≤ 0.01 between 3- and 10-day-old.

** p<0.01 between 7- and 10-day-old

### Functional characteristics of islets from various ages of neonatal pigs

**Figure 2:**
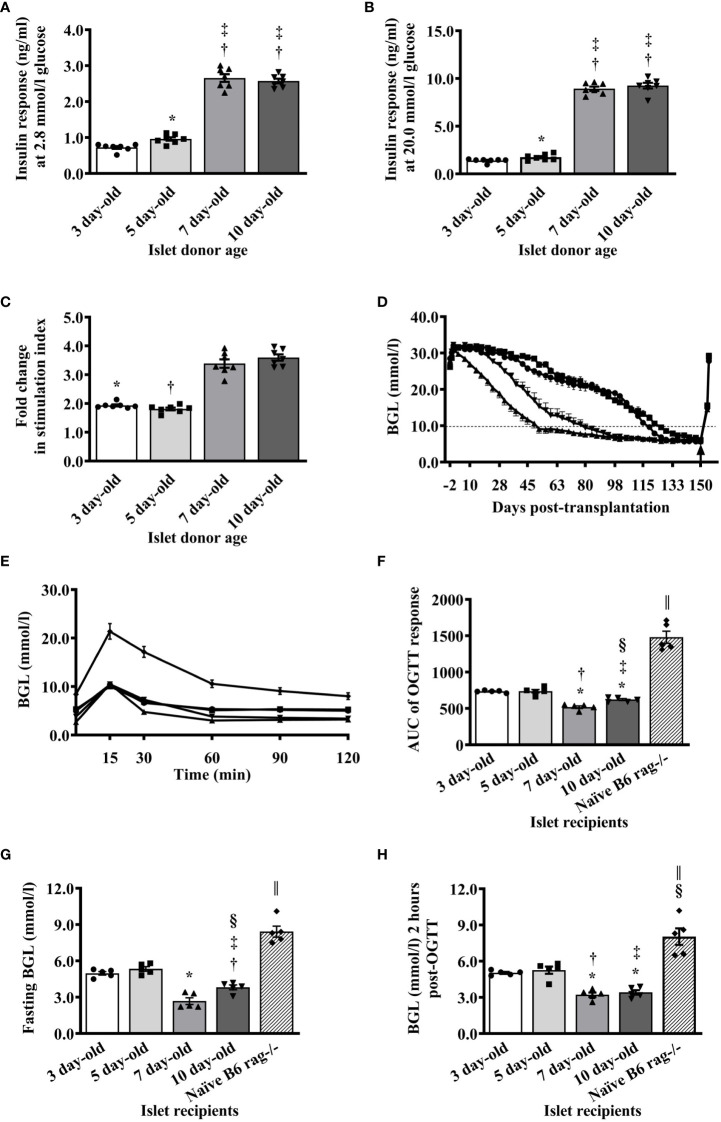
Functional characteristics of islets isolated from neonatal pigs of various ages. **(A)** Responses of islets as measured by the amount of insulin detected in the supernatant after stimulation of islets with 2.8 mmol/l glucose using in vitro glucose stimulation assay (*p <0.005 between 3- and 5-day-old; ^†^p <0.0001, 3- vs. 7- and 10-day-old; ‡p <0.0001, 5- vs. 7- and 10-day-old). **(B)** Amount of insulin detected in the supernatant after stimulation of islets with 20 mmol/l glucose (*p <0.05 between 3- and 5-day-old; ^†^p <0.0001, 3- vs. 7- and 10-day-old; ^‡^p <0.0001, 5- vs. 7- and 10-day-old). **(C)** Insulin response expressed as stimulation index of islets from various ages of neonatal pigs to glucose stimulation in vitro (*p <0.0001, 3-day-old vs. 7- and 10-day-old; ^†^p <0.0001, 5- vs. 7- and 10-day-old). Data from 3-, 5-, 7-, and 10- (n=7 for each group) day-old pigs are shown. **(D)** Blood glucose levels of B6 rag-/- mouse recipients before and after transplantation of 2000 I.E. from various ages of neonatal pigs. Recipients of NPI are shown as circle symbol for 3-day-old (n=40), square symbol for 5-day-old (n=30), triangle symbol for 7-day-old (n=40), and reverse triangle symbol for 10-day-old (n=20). Arrow indicates the time when the kidney that contained the islet xenograft was removed from each mouse recipient. Normoglycemia and hyperglycemia are defined as <10mmol/l and >10mmol/l, respectively. **(E)** Responses of non-transplanted or transplanted B6 rag-/- mice with 2000 I.E. from various ages of neonatal pigs during oral glucose challenge at <130 days post-transplantation. Circle, square, triangle, reverse triangle, and diamond symbols represent recipients of 3-, 5-, 7-, and 10-day-old pigs, and non-transplanted B6 rag-/- mice (n=5 for each group), respectively. **(F)** Responses expressed as AUC of non-transplanted or transplanted B6 rag-/- mice with islets from various ages of neonatal pigs during oral glucose challenge at >130 days post-transplantation. *p <0.0001, 3-day-old vs. 7- and 10-day-old; †p ≤0.001 between 3- and 10-day-old. ‡p ≤0.0005 between 5- and 10-day-old; §p ≤0.01 between p 0.001 between 3- and 10-day-old; ǁp <0.0001 between non-transplanted B6 rag-/- and other groups. **(G)** Fasting blood glucose levels of non-transplanted B6 rag^-/-^ mice and those that were transplanted with islets from various ages of neonatal pigs. *p <0.0001, 3- or 5-day-old vs. 7-day-old; ^†^p ±0.001 between 3- and 10-day-old. ^‡^p ±0.0005 between 5- and 10-day-old; §p ±0.01 between 7- and 10-day-old; ǁp <0.0001 between non-transplanted B6 rag-/- and other groups. **(H)** Blood glucose levels of non-transplanted B6 rag-/- mice and those that received islets from various ages of neonatal pigs 2 hours post-glucose challenge. *p <0.0001, 3- vs. 7- or 10-day-old; ^†^p <0.0005 between 5- and 7- day-old; ^‡^p < 0.001 between 5- and 10- day-old; §p <0.01, 3- and 5- day-old vs. non-transplanted B6 rag-/-; ǁp <0.0001, 7- and 10-day-old vs. non-transplanted B6 rag-/-. Data from recipients of 3-, 5-, 7-, and 10-day-old pigs, and non-transplanted B6 rag-/- mice (n=5 for each group) are shown.

When islets from various ages of pigs were challenged with low (2.8 mmol/l) and high (20 mmol/l) glucose *in vitro*, there was a significant difference in the response of islets from 5-day-old compared to islets from 3-day-old pigs. Islets from 7- and 10-day-old pigs responded with significantly more insulin compared to islets from 3- and 5-day-old pigs (**Figures 2A, B**). A similar pattern was detected when the stimulation index (SI) from different groups was calculated (**Figure 2C**).

The ability of islets from various ages of pigs to reverse hyperglycemia in diabetic B6 *rag-/-* mice was evaluated. Mice that received 2000 I.E. from 7-day-old pigs achieved normoglycemia on average at earlier time points post-transplantation (day 52) compared to the mice that received 2000 I.E. from 10-, 3-, and 5-day-old pigs (day 80, 119, and 122, respectively, **Figure 2D**). At the end of the follow-up period (>150 days post-transplantation), all mice became diabetic when the kidney with the islet xenograft (**Supplementary Figure 2** and **Figure 2D**) was removed indicating that normoglycemia was maintained by the islet transplant (**Figure 2D**). Randomly selected mice in each group were able to lower their blood glucose levels regardless of the age of the pig islet donor to much lower values compared to the naïve (normoglycemic, non-transplanted) B6 *rag-/-* mice post-oral glucose challenge (**Figure 2E**). However, when the area under the curve (AUC) was calculated, there were significant differences in the response of recipients of 3- and 5- compared to the recipients of 7- and 10-day-old pig islets (**Figure 2F**). There was also a significant difference in the response of islet recipients compared to the response observed in naïve non-transplanted mice. A similar pattern was detected in the fasting blood glucose levels before (**Figure 2G**) and 2 hours after the glucose challenge of recipient mice (**Figure 2H**).

### Islets from 7-day-old pigs are more efficient in reversing hyperglycemia than islets from 3-day-old pigs

**Figure 3:**
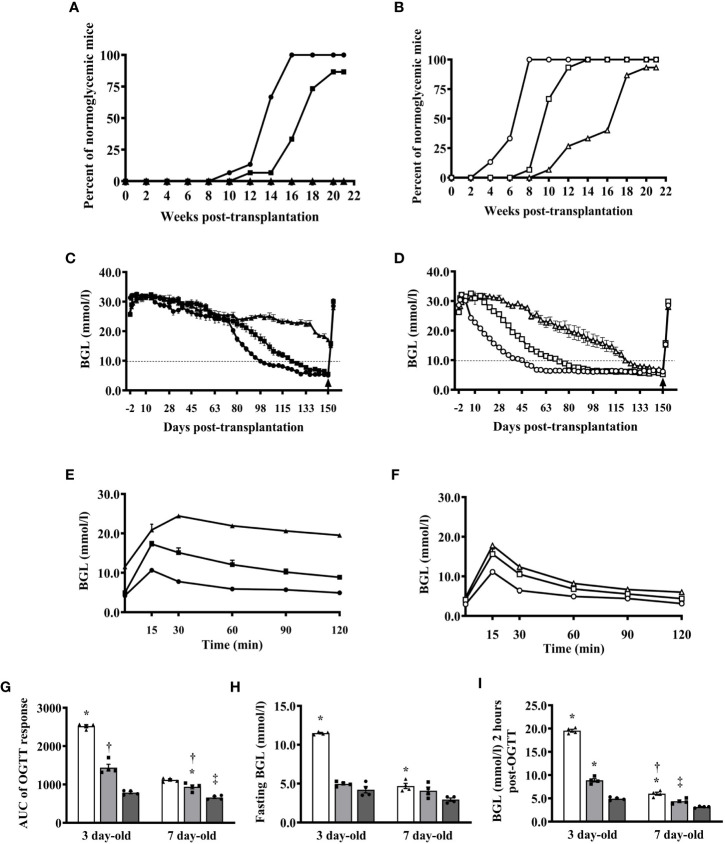
Reversal of diabetes in B6 rag-/- mouse recipients of various numbers of islets from 3- and 7-day-old pigs. **(A)** Proportions of mice transplanted with 500, 1000, and 2000 I.E. (n=15 per group) from 3-day-old pigs that achieved normoglycemia at various time points after transplantation. **(B)** Proportions of mice transplanted with 500, 1000, and 2000 I.E. (n=15 per group) from 7-day-old pigs that achieved normoglycemia at various time points post-transplantation. **(C)** Blood glucose levels of mice transplanted with various numbers of islets from 3-day-old pigs (n=15 per group). **(D)** Blood glucose levels of mice transplanted with various numbers of islets from 7-day-old pigs (n=15 per group). **(E)** Responses of B6 rag-/- mouse recipients of various numbers of islets from 3-day-old pigs after oral glucose challenge at >130 days after transplantation (n=4 per group). **(F)** Responses of mice transplanted with various numbers of islets from 7-day-old pigs post-oral glucose challenge (n=4 per group). Black and white symbols represent mice that received 500 (triangle), 1000 (square), and 2000 (circle) I.E. from 3- and 7-day-old pigs, respectively. **(G)** Calculated AUC responses of B6 rag-/- mouse recipients of islets from 3- and 7-day-old pigs after oral glucose challenge at >130 days post-transplantation. *p <0.0001, 500 I.E. vs. 1000 I.E and 2000 I.E.; †p <0.0005 between 1000 I.E. and 2000 I.E for 3-day-old and *p <0.005 between 1000 I.E. and 2000 I.E.; †p <0.05 between 500 I.E. vs. 1000 I.E.; ‡p <0.0001 between 500 I.E. and 2000 I.E. for 7-day-old. **(H)** Fasting blood glucose levels of B6 rag-/- mouse recipients of islets from 3- or 7-day-old pigs. *p <0.0001 between 500 I.E. and 1000 I.E or 2000 I.E. for 3-day-old and *p <0.005 between 500 I.E. and 2000 I.E. for 7-day-old. **(I)** Blood glucose levels of B6 rag-/- mouse recipients of islets from 3- or 7-day-old pigs 2 hours post-glucose challenge. *p <0.0001, 500 I.E. and 1000 I.E. vs. 2000 I.E. for 3-day-old and *p <0.01, between 500 I.E. vs. 1000 I.E.; †p <0.0001 between 500 I.E. and 2000 I.E.; ‡p ≤ 0.005 between 1000 I.E. and 2000 I.E. White (triangle), gray (square), and dark gray (circle) bars (symbol) represent data from B6 rag-/- mice that were transplanted with 500, 1000, and 2000 I.E., respectively (n=4 for each group).

Since our previous studies utilized islets from 1- to 3-day-old pigs and based on the results above showing that islets from 7- and 10-day-old pigs responded better to glucose stimulation *in vitro* and *in vivo* than islets from 3- and 5-day-old pigs and that 7-day-old pigs can provide a significantly better yield of islets than 10-day-old pigs, we now focus our study in comparing islets from 3- and 7-day-old pigs. We first compared the ability of various numbers of islets from 7-day-old pigs to that of 3-day-old pigs in reversing the diabetic state of B6 *rag-/-* mouse recipients. Mice that received 500 I.E. from 3-day-old pigs showed a reduction in their blood glucose levels but remained in the diabetic state until the end of the study (>20 weeks post-transplantation, **Figures 3A, C**). Four of these mice died at 6 weeks post-transplantation, due to poor health related to diabetes. Two of the recipients of 1000 I.E. from 3-day-old pigs died at 3 weeks post-transplantation. One of the recipients of 1000 and 2000 I.E. from 3-day-old pigs achieved normoglycemia at 12 and 10 weeks post-transplantation, and all remaining recipients achieved normoglycemia at 20 and 16 weeks post-transplantation, respectively (**Figures 3A, C**). In contrast, only one mouse recipient of 500 I.E. from 7-day-old pigs died at day 35 post-transplantation. One, one, and two mice that received 500, 1000, or 2000 I.E. from 7-day-old pigs achieved normoglycemia at 10, 8, and 4 weeks post-transplantation, respectively (**Figures 3B, D**). The remaining mice in these groups achieved normoglycemia at 20 (500 I.E.), 14 (1000 I.E.), and 8 (2000 I.E.) weeks post-transplantation (**Figure 3D**). All recipients of islets from 3-day-old pigs, except those mice that received 500 I.E., were able to return to their normoglycemic state after challenge with oral glucose (**Figure 3E**). Recipients of 2000 I.E. were more efficient in achieving normoglycemia than recipients of 1000 I.E. In contrast, all mice that received 500, 1000, and 2000 I.E. from 7-day-old pigs were able to reduce blood glucose to normal levels after the glucose challenge (**Figure 3F**). The calculated AUC of the responses observed in recipients of various amounts of islets from 3- and 7-day-old pigs significantly decreased as more islets were transplanted (**Figure 3G**). A similar pattern was observed in fasting and at 2 hours post-glucose challenge (**Figures 3H, I**)

### Reduced Kv and increased Ca^2+^ channel activity in islets from 7-day-old pigs

**Figure 4:**
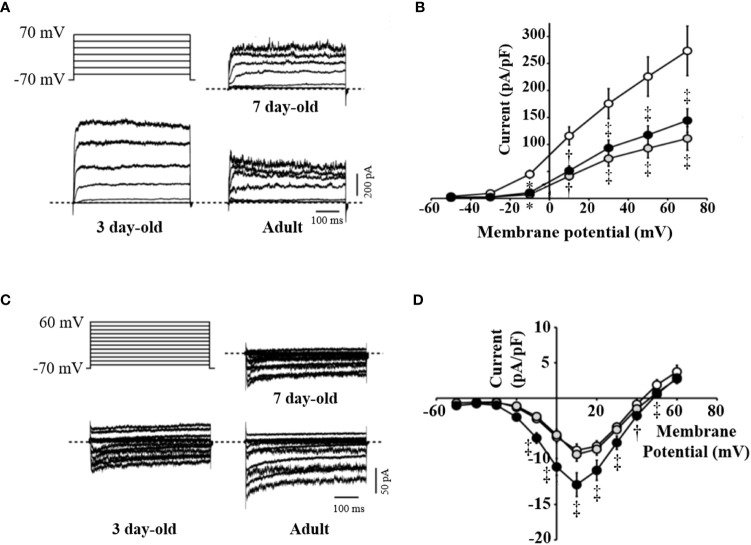
Activity of voltage-dependent K^+^ and Ca^2+^ channels in beta cells of 3-, 7-day-old neonatal and adult pig islets. Voltage-dependent K^+^ (Kv) channels or voltage-dependent Ca^2+^ channels (VDCCs) were recorded in response to a series of membrane depolarizations from a holding potential of -70 mV. **(A)** Stimulation protocol and representative traces of Kv currents from islet beta cells of 3- (n=17), 7-day-old neonatal (n=17) and adult pigs (n=10). **(B)** Averaged current-voltage relationship of Kv channel activity in these groups. **(C)** Stimulation protocol and representative traces of VDCC currents from islet beta cells of 3-day-old (n=21), 7-day-old neonatal (n=22) and adult pigs (n=19). **(D)** Mean current-voltage relationship of VDCC activity in these groups. ^*^p <0.001, ^†^p <0.01, ^‡^p <0.05 compared with islets from 3-day-old pigs. White, gray, and black circles represent data from 3-day-old, 7-day-old, and adult pig islets, respectively.

VDCCs are critical mediators of beta cell electrical activity, Ca^2+^ entry, and subsequent insulin release in response to glucose (26). Reductions in Kv channel current are associated with increases in glucose-stimulated electrical activity and insulin secretion (27), while elevated Ca^2+^ entry through VDCCs will likewise increase insulin secretion. The Kv channel activity was decreased by 59% in beta cells from 7-day-old compared to beta cells from 3-day-old pigs (**Figures 4A, B**). The Kv current density in islets from 7-day-old pigs was similar to that seen in the adult pig beta cells. Furthermore, we observed no difference in VDCC activity between islets from 3- and 7-day-old pigs, which were both approximately 35% reduced compared with that typically observed in adult pig beta cells (**Figures 4C, D**). Thus, the improved function of islets from 7-day-old pigs likely cannot be attributed to increased Ca^2+^ channel function but may in part be due to reduced Kv channel activity, which could lead to an increase in electrical function.

### Islets from 7-day-old pigs may be more immunogenic than islets from 3-day-old pigs

**Figure 5:**
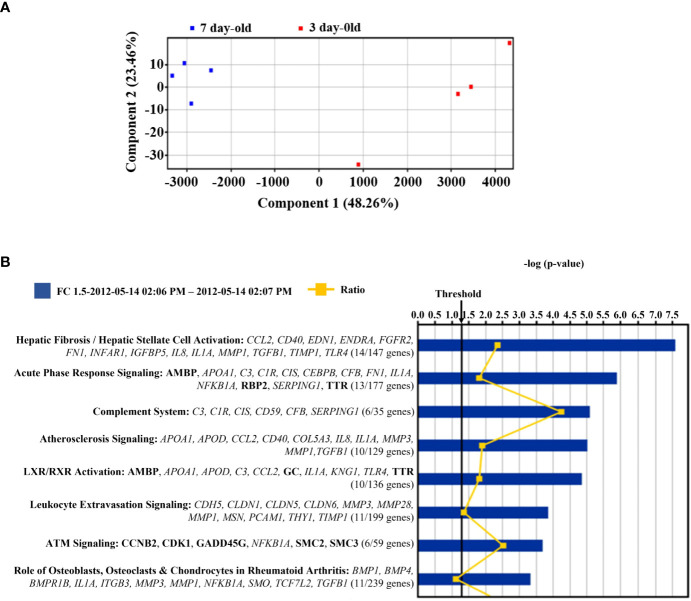
Microarray analysis of islets from 3- and 7-day-old pigs. **(A)** Principal component analysis of eight samples of islets isolated from 3- and 7-day-old pigs. The red and blue squares denote islets from 3-day-old and 7-day-old pigs, respectively. The x-axis and y-axis are a visual representations of unidentified vectors that separate the samples. This figure displays the difference between islets from 3- and 7-day-old pigs. **(B)** Canonical pathways represented in the differentially expressed genes between islets from 3- and 7-day-old pigs. The p-value is calculated by Fisher’s Exact Test and is displayed in blue as a –log with more significant pathways being higher on the scale. The threshold represents a p-value of 0.05. The orange line displays the ratio of genes that are significantly expressed between the groups and the total number of genes in each pathway. This ratio is also written under each pathway. The genes from the significance analysis are displayed directly under each pathway. The non-bold genes denote up-regulation, and the bold genes denote down-regulation.

The microarray principal component analysis of all samples without any *a priori* sample classification demonstrated a robust differentiation between the islets from 3-day-old compared to islets from 7-day-old pigs (**Figure 5A**). Principal component one alone accounted for around 50% of the difference between the two islet sample groups. Out of the 20,056 probe sets that passed the initial filtering step 4,048 probe sets were differentially expressed between islets from 3- and 7-day-old pigs (adjusted p-value <0.05). Using a fold change cut-off of ≥1.5 or 2, respectively, out of significantly different probe sets 686 or 236 were up-regulated and 624 or 135 were down-regulated in islets from 7-day-old compared to 3-day-old pigs, respectively. Matrix Metallopeptidase (*MMP*) 3 (9.1-fold), Alveolar Macrophage Chemotactic Factor 2 (*AMCF-II*, 7.7-fold), *MMP1* (7.5-fold), and Prostaglandin-Endoperoxide H Synthase 2 (*PGHS-2*, 6.8-fold) were the most up-regulated genes in islets from 7-day-old compared to 3-day-old pigs (**Table 2**). We also examined genes that may be involved in insulin secretion and found that Calveolin 1 (*CAV1*, 2.9-fold), Annexin A1 (*ANXA1*, 2.7-fold), Transforming Growth Factor beta 1 (*TGFβ1*, 2.3-fold), and Apolipoprotein A1 (*APOA1*, 2.1-fold) were up-regulated in islets from 7-day-old compared to islets from 3-day-old pigs (**Table 2**). The genes that were found to be down-regulated in islets from 7-day-old compared to islets from 3-day-old pigs comprise Adenylate Kinase 7 (*AK7*, 2.9-fold), Group-specific component (*GC*, 2.6-fold), citrullinated inhibitor of DNA binding 1 (*CITED1* 2.6-fold), never in mitosis gene A (NIMA)-related expressed kinase 4 (*NEK4*, 2.5-fold), Contactin 4 (*CNTN4*, 2.5-fold), and Neurogenic Differentiation 1 (*NEUROD1*, 2.4-fold) (**Table 3**).

**Table 2:** Most up-regulated (+) genes in islets from 7-day-old pigs compared to islets from 3-day-old pigs.

Gene Symbol	Gene Function	Fold Change	p-Value	Corrected p-Value
***MMP3***	Tissue remodeling and embryonic development, pleiotropic roles by modulating inflammatory mediators such as cytokines and chemokines, regulating leukocytes influx (28–30)	+ 9.1	0.0036	0.028
***AMCF-II***	Chemotactic activity for pig neutrophils (31, 32)	+ 7.7	8.6E-5	0.0060
***MMP1***	Tissue remodeling and embryonic development (28–30)	+ 7.5	8.6E-4	0.013
***PGHS-2***	Increases the capacity of human alveolar macrophage and blood monocyte to generate prostaglandins after exposure to lipopolysaccharide (32)	+ 6.8	3.2E-4	0.0088
***CAV1***	Normally present in beta cells, where it participates in the regulation of insulin secretion CAV1 was also shown to regulate insulin receptor internalization and ERK activation in human and mouse beta cells, implicating CAV1 as a relevant player in beta cell physiology (33–35).	+ 2.9	1.3E-4	0.000582
***ANXA1***	Enhances glucose-stimulated insulin secretion (36).	+ 2.7	1.2E-3	0.001703
***TGFβ1***	Plays diverse roles in the development, proliferation, apoptosis, dedifferentiation, and function of islet beta cells in humans and rodents (37–39).	+ 2.3	1.3E-4	0.000856
***APOA1***	Improves insulin secretion, increases nuclear localization of PDX1 and the levels of proinsulin, increases insulin granules (40).	+ 2.1	2.7E-4	0.000691

**Table 3:** Most down-regulated (-) genes in islets from 7-day-old pigs compared to islets from 3-day-old pigs.

Gene Symbol	Gene Function	Fold Change	p-Value	Corrected p-Value
***AK7***	Regulates the homeostasis of adenine nucleotide composition within cellular compartments to maintain cellular energy homeostasis and control the ATP/ADP ratio in the microenvironment of K-ATP channels and in the regulation of the actual rate of insulin secretion (41, 42)	- 2.9	08.6E-5	0.0060
***GC***	Transportation of Vitamin D metabolites binding of fatty acids, and modulation of immune responses (43–46)	- 2.6	2.9E-4	0.0084
***CITED1***	This gene encodes a member of the CREB-binding protein/p300-interacting transactivator with Asp/Glu-rich C-terminal domain (CITED) family of proteins. CITED1 plays an important role in regulating trophoblast lineage specification by activating the BMP signaling pathway (47)	- 2.6	5.5E-6	0.0029
***NEK4***	Mediates cell migration and metastasis by promoting the epithelial-mesenchymal transition process in cancer cells and regulates E-cadherin (48)	- 2.5	1.6E-6	0.0028
***CNTN4***	Detailed function is not described	- 2.5	2.1E-5	0.0042
***NEUROD1***	Regulates islet beta cell function and mediates glucose- regulated insulin gene transcription during prenatal development and in the adult (49)	- 2.4	3.6E-5	0.0045

A Gene Ontology (GO) analysis was performed on up-regulated probe sets in islets from 7-day-old pigs that passed the 2-fold cut-off. The GO analysis identified chemokine/cytokine activity and receptor binding (including *CCL2*, *CXCL2*, *IL8*, *AMCF-II*, and *CXCL14*) as significant molecular functions (adjusted p-value <0.01). Corresponding to these molecular functions the GO analysis computed inflammatory response as a significant biological process (*CCL2*, *IL8*, *IL1A*, *AMCF-II*, *BMPR1B*, *TLR4*, and *TGFB1*). The pathway analysis using Ingenuity software on all genes differentially expressed and changing >1.5-fold identified the canonical pathways as shown in **Figure 5B**. The most significant pathways are hepatic fibrosis, acute phase response signalling, and the complement system. In almost all the pathways, the significant genes were up-regulated (on islets from 7-day-old pigs) except for Ataxia-telangiectasia-mutated (ATM) signalling, which has more down-regulated genes. Most of the selected genes are inflammation associated.

Islets from 7-day-old contained significantly more CD4^+^ and CD8^+^ T cells, B cells, macrophages, and leukocytes compared to islets from 3-day-old pigs. Adult pig PBMCs, as a control, had significantly more immune cells except macrophages (SWC9) compared to islets from 3- and 7-day-old pigs (**Table 4** and **Supplementary Figure 1**).

**Table 4:** Proportions of immune cells in islets from 3- and 7-day-old pigs.

Group	n	CD4	CD8	sIg	SWC9	CD45
**3-day-old**	6	0.81 ± 0.08	0.29 ± 0.06	0.28 ± 0.06	2.42 ± 0.25	0.72 ± 0.04
**7-day-old**	4	2.10 ± 0.23*	1.09 ± 0.11*	0.80 ± 0.11^†^	4.20 ± 0.58^‡^	1.08 ± 0.10^†^
**Adult pig PBMCs**	3	21.49 ± 5.24* ^‖^	32.07 ± 3.51* ^§^	14.24 ± 1.72* ^§^	2.56 ± 1.11	96.18 ± 0.09* ^§^

Pig islets were dissociated into single cells following our standard protocol as described. Following dissociation, 0.5×10^6^ cells were incubated with antibodies to specific immune cell markers. The percentage of immune cells expressing the markers was analyzed by flow cytometry. Adult pig PBMCs were used as a positive control.

*p ≤0.0005 between islets from 3- vs. 7-day-old pigs or adult pig PBMCs.

^†^p ≤0.005 between islets from 3- vs. 7-day-old pigs.

^‡^p ≤0.05 between islets from 3- vs. 7-day-old pigs.

^§^p ≤ 0.0005 islets from 7-day-old vs. adult pig PBMCs.

^‖^p ≤0.01 islets from 7-day-old vs. adult pig PBMCs.

### Islets from 7-day-old pigs are more efficient in retrieving their function post-hypoxia/reoxygenation stress

**Figure 6:**
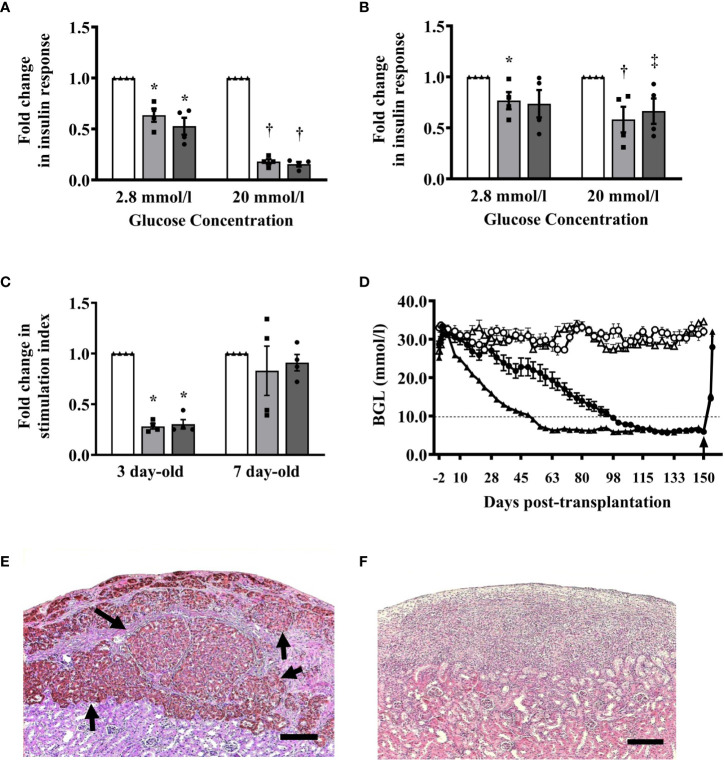
**(A–C)** Insulin responses of islets after exposure to stress induced by hypoxia and reoxygenation. **(A)** Insulin response of islets from 3-day-old pigs (n=4) when challenged with 2.8 mmol/l (0.63 ± 0.64, ^*^p ≤0.001; 0.53 ± 0.08, ^*^p ≤0.001) and 20 mmol/l glucose concentrations (0.18 ± 0.02, ^†^p <0.0001; 0.15 ± 0.02, ^†^p <0.0001) after exposure to hypoxia and reoxygenation, respectively compared to untreated islets. **(B)** Insulin response of islets from 7-day-old pigs (n=4) when challenged with 2.8 mmol/l (0.77 ± 0.08, ^*^p <0.05; 0.74 ± 0.14) and 20 mmol/l glucose (0.58 ± 0.13, ^†^p <0.05; 0.67 ± 0.12, ^‡^p <0.05) after exposure to hypoxia and reoxygenation, respectively compared to untreated islets. **(C)** Stimulation index response of islets from 3- and 7- day-old pigs after exposure to hypoxia (0.28 ± 0.03, ^*^p <0.0001; 0.83 ± 0.24) and reoxygenation (0.30 ± 0.046, ^*^p <0.0001; 0.91 ± 0.08), respectively. Responses were expressed as fold-change in insulin secretion after exposure to hypoxia (gray bars, square symbols) and reoxygenation (dark gray bars, circle symbols) when compared to islets that were not exposed to the same stressors (white bars, triangle symbols). **(D–F)** Blood glucose levels and morphology of islet xenografts from B6 mice transplanted with 2000 I.E. from 3- or 7-day-old pigs. **(D)** All B6 mice (n=10 in each group) transplanted with islets from 3-day-old (black circle) or 7-day-old (black triangle) pigs treated with a combination of anti-LFA-1 and anti-CD154 mAbs achieved and maintained normoglycemia for >150 days post-transplantation while untreated B6 mice (n=5 in each group) transplanted with islets from 3-day-old (white circle) or 7-day-old (white triangle) neonatal pigs remained diabetic throughout the study period. The arrow shows the time when survival nephrectomy was performed on recipients. **(E)** Representative islet graft from B6 mice transplanted with islets from 7-day-old pigs treated with combined anti-LFA-1 and anti-CD154 mAbs at >150 days post-transplantation. Brown-stained structures (arrow) represent intact islets with insulin-positive beta cells. **(F)** Representative islet graft from untreated B6 mice transplanted with islets from 7-day-old pigs at >150 days post-transplantation. There were no intact islets present at the graft site. Scale bar represents 100 μm.

We compared the *in vitro* response of islets from 3- and 7-day-old pigs after exposure to hypoxia (1% oxygen) and reoxygenation (normal incubation condition)-induced stress. After exposure to hypoxia, a significant decrease in insulin response of islets from 3-day-old pigs compared to untreated islets was observed when challenged with low (2.8 mmol/l) and high (20 mmol/l) glucose concentrations (**Figure 6A**). The changes in insulin response of hypoxia-treated islets from 7-day-old pigs when challenged with low glucose were not significant when compared to untreated islets. However, the insulin response of these islets was significantly lower compared to untreated islets when challenged with high glucose (**Figure 6B**). The changes in SI response of islets from 3- and 7-day-old pigs after exposure to hypoxia compared to untreated islets were also significant, but not after reoxygenation of islets from 7-day-old pigs when compared to untreated islets (**Figure 6C**).

### A combination of anti-LFA-1 and anti-CD154 mAbs prevents the rejection of islets from 7-day-old pigs

Previously, we demonstrated that a combination of anti-LFA-1 and anti-CD154 mAbs induced tolerance to 3-day-old pig islet xenografts in B6 mice (7, 9). We confirmed these results and found that B6 mouse recipients of 2000 I.E. from 3-day-old pigs treated with combined anti-LFA-1 and anti-CD154 mAbs achieved normoglycemia on average at 93 days post-transplantation. In addition, mice that received islets from 7-day-old pigs achieved normoglycemia at 52 days post-transplantation (**Figure 6D**). These mice maintained their normoglycemic state for >150 days post-transplantation. Examination of the islet xenografts revealed abundant intact islets that stained positive for insulin with few mononuclear cells surrounding the islets (**Figure 6E**). None of the untreated mouse recipients achieved normoglycemia post-transplantation (**Figure 6D**) and there were no islets detected at the graft site in these mice indicating complete rejection of the islet xenografts (**Figure 6F**).

## Discussion

Neonatal pigs can potentially provide an unlimited source of islets for transplantation into patients with type 1 diabetes and pig islet xenotransplantation may become the treatment of choice for type 1 diabetes in the near future. As such, the International Xenotransplantation Association has developed a consensus statement on conditions for undertaking clinical trials of pig islet xenotransplantation (17, 18). Based on this consensus, a key component for ensuring control of a validated islet manufacturing process is the use of pre-defined acceptance criteria for the pancreas (17, 19). One criterion should warrant that only suitable donor pancreas with maximal potential for yielding numbers of islets are used for islet manufacturing. In terms of neonatal pig donors, it remains unknown at what age of pigs would be ideal to provide a maximum yield of functional islets. Thus, this study evaluated the morphological and functional characteristics of islets from various ages of neonatal pigs to provide guidance in addressing this question. Although numerous studies have characterized and compared islets from various ages of pigs, these studies were mostly focused on *in vitro* characterization (2, 50, 51) and the *in vivo* function, as well as the susceptibility of islets from various ages of pigs to immune-mediated rejection, has not been studied.

Since our previous studies to date utilized islets from 1 to 3-day-old pigs, we were interested to find out whether our results could be improved in terms of islet yield and potency when older neonatal pigs are used as the source of islets. Our results suggest that 7-day-old pigs could provide a consistent amount of tissues as part of a well-controlled manufacturing process. Using our method of islet isolation, we found that the pancreas of 10-day-old pigs produces a significantly less number of islets compared to the pancreas of 3-, 5-, and 7-day-old pigs. This is reflected in the results we obtained for the total DNA content measured from these islets. However, islet beta cells from 7- and 10-day-old pigs responded better in terms of the amount of insulin they produced after stimulation with glucose compared to beta cells from 3- and 5-day-old pigs. These results confirmed previous findings which indicate that the beta cells in older pigs are more responsive to glucose challenges *in vitro* (50). Our *in vivo* results further supported these findings when the ability of various amounts of islets from 3- and 7-day-old pigs in reversing diabetes in mice was compared. Islets from 7-day-old pigs were more efficient than islets from 3-day-old pigs at reversing diabetes in both immune-deficient and immune-competent mice. These findings may be partly due to beta cells from 7-day-old pigs having reduced Kv channel activity, which could lead to an increase in electrical function or responsiveness to glucose stimulation.

Further, previous findings have shown that the membrane integrity of pig islets was age-dependent (42) and our novel finding may only be the beginning of the identification of integral membrane components that differ between various ages of pigs. For example, *CAV1* gene, which encodes for caveolin 1, the main protein component of the caveolae plasma membranes found in most cells including beta cells, was also found to be up-regulated in islets from 7-day-old pigs compared to islets from 3-day-old pigs. Calveolin 1 was reported to participate in the regulation of insulin secretion, and recently, it was also shown to regulate insulin receptor internalization and ERK activation in human and mouse beta cells, implicating calveolin 1 is an important player in beta cell physiology (33–35). *ANXA1* gene, which codes for annexin A1 protein, a membrane-localized protein that binds phospholipids and has anti-inflammatory activity has been shown to be a key modulator of mesenchymal stromal cell-mediated improvements in islet function (36). Treatment of mouse islets with annexin A1 resulted in an increase in high glucose-stimulated insulin secretion. It is interesting to note that the expression of *TGFB1* gene was also up-regulated in islets from 7-day-old compared to islets from 3-day-old pigs. TGFβ1 has been shown to play diverse roles in the development, proliferation, apoptosis, dedifferentiation, and function of islet beta cells in humans and rodents (37–39). For example, rat islets treated with TGFβ1 stimulates insulin secretion in low glucose condition but has no effect in high glucose condition (52). While in mouse islets, treatment with TGFβ1 resulted in decreased expression of genes involved in insulin synthesis, processing, and secretion (53). Finally, *APOA1* gene, which encodes for ApoA1 protein has also been shown to improve glucose-stimulated insulin secretion of mouse islets and a beta cell line (40). The authors reported that this effect appeared to be due to an increased reservoir of insulin granules at the cell membrane, as confirmed by confocal and transmission electron microscopy (40). Moreover, it was found that ApoA1 induced pancreatic and duodenal homeobox 1 (PDX1) shuttling from the cytoplasm to the nucleus, resulting in the subsequent increase in the proinsulin processing enzyme protein convertase 1 (PC1/3) (40). The role of TGFβ1 and ApoA1 in pig islet insulin synthesis, processing, and secretion during postnatal period remains to be elucidated.

Differential expression data also revealed that six genes were most down-regulated in islets from 7-day-old compared to islets from 3-day-old pigs. Of most interest is the AK7 gene that encodes the KAD7 protein. KAD7 belongs to AK ubiquitous phosphotransferases that play a role in controlling the ATP/ADP ratio in the microenvironment of K/ATP channels and in the regulation of the actual rate of insulin secretion. Glucose-induced decrease in AK-catalyzed phosphotransferase results in a localized increase in ATP and a decrease in ADP levels promoting K/ATP channel inhibition, Ca^2+^ influx, and insulin secretion (41, 42). The bioenergetics pathways that couple metabolism to insulin secretion during the postnatal period is another area in porcine islet biology that offers opportunities for future studies.

Our results also show higher proportions of CD4^+^ and CD8^+^ T cells, B cells, and macrophages/leukocytes (SWC9) in islets from 7-day-old pigs, which indicates that these islets may have a tendency to be more immunogenic than islets from 3-day-old pigs. This could be due to the difference in the maturation state of the immune system of neonatal pigs. For example, SWC9 is a cell surface antigen expressed only on mature pig macrophages. Furthermore, based on the microarray data islets from 7-day-old pigs showed significantly higher expression of *MMP-3*, *AMCF-II*, *MMP-1*, and *PGHS-2* genes which encode proteins involved in inflammation. However, the role of these genes and encoded proteins in the postnatal development of islets remains an open area of research. It is possible that the over-activation of these genes and the increased immune cell influx in islets from 7-day-old pigs is associated with pancreatic tissue development. For example, the *AMCF-II* gene encodes for AMCF-II protein, a molecule produced by pig alveolar macrophages, and has potent chemotactic activity for pig neutrophils (31, 32). This could be attributed to their significantly high expression of *MMP3* gene which encodes for MMP3 enzyme that degrades various molecules involved in connective tissue remodelling (28). MMP3 can also activate other MMPs, and modulate inflammatory mediators and leukocyte influx (29, 30). Collectively these results may predispose islets from 7-day-old pigs to be more susceptible to innate immunity in the immediate post-transplant period. A study by Smith et al. reported transcriptome data on islets from various ages of pigs and demonstrate that transcriptome analysis was consistent with physiological assessments of the islets (50). In our study, we were particularly interested in the immune component of transcriptome analysis and speculated that changes in the physiology of islets may predispose them to inflammatory response and/or immune-mediated destruction post-transplant. Future studies examining the intragraft events at various time points post-transplantation and/or concentration of porcine insulin or c-peptide in the blood of mice over time could determine whether the observed differences in the time to normoglycemia between mouse recipients of islets from 3- vs. 7-day-old pigs are mainly due to islet beta cell development or due to differences in the protection of islets from inflammatory injury.

Although it was previously demonstrated that NPI (1-3-day-old) exhibits natural resistance to hypoxia-induced apoptosis (4), we found that islets from 7-day-old pigs show an improved capacity to recover from stress-induced hypoxia during islet isolation procedure and at early time points post-transplantation compared to islets from 3-day-old pigs. Hypoxia-induced malfunction and apoptosis in beta cells during isolation and at early time points post-transplantation have been reported in human and rodent islet transplantation, mandating the need for large numbers of islets to be transplanted (54–58). After intra-portal islet infusion, this hypoxic stress is extended for up to two weeks until revascularization and consequently, reoxygenation occurs, which in turn triggers reoxygenation stress and/or injury (59–62). We showed that islets from 7-day-old pigs are more capable of recovering their function after exposure to hypoxia- and reoxygenation-induced stress than islets from 3-day-old pigs exposed to the same conditions.

Despite the potential vulnerability of islets from 7-day-old pigs to immune-mediated rejection post-transplantation, transient administration of combined anti-LFA-1 and anti-CD154 mAb therapy promoted their survival similar to what we found in islets from 3-day-old pigs (7, 9). This combined monoclonal antibody therapy has the advantage of selectively targeting cells and pathways of the immune system namely LFA-1/ICAM-1 and CD40/CD154 co-stimulatory pathways as opposed to general immunosuppression that is characteristics of conventional immunosuppressive drugs. In addition, these monoclonal antibodies have been shown to be effective at promoting the survival of porcine islets, particularly when given short-term as opposed to chronic administration of conventional immunosuppressive drugs.

In conclusion, we showed that islets from 7-day-old pigs could offer some advantages over 3-day-old pigs and the immunogenicity of these islets could be overcome by the treatment with a combination of biologic agents that block co-stimulatory pathways. These findings may have implications for attempts to refine the most appropriate age of neonatal pigs for islet isolation and transplantation and similar strategies could be employed in the identification of optimal pancreas donors to further progress the field of islet xenotransplantation.

## Data availability statement

The original contributions presented in the study are publicly available. This data can be found here: https://www.ncbi.nlm.nih.gov/geo/query/acc.cgi?acc=GSE220256.

## Ethics statement

The animal study was reviewed and approved by the University of Alberta Animal Care and Use Committee under protocol number AUP326.

## Author contributions

GR and HA designed the project. HA conducted the porcine islet isolation and transplantation, *in vitro* assays, flow cytometry and immunostaining assays, chemical induction of diabetes, monitoring the blood glucose levels of mice, induction of hypoxia, and reoxygenation stress, mAbs treatment, contributed to data analysis, and preparation of the manuscript. QR assisted in porcine islet isolation, chemical induction of diabetes, monitoring the blood glucose levels of mice, and data analysis. NG conducted an analysis of data and preparation of the manuscript. AS and PD designed and conducted studies on K^+^ and Ca^+^ channels on islet beta cells and data analysis as well as contributed to the preparation of the paper. DG and TM analyzed the microarray data, and statistical analysis, and contributed to the preparation of the paper. LG provided the adult pig islets and contributed to the preparation of the paper. RR contributed critical reagents and helped design the experiments. GR wrote the paper, designed the experiments, and supervised the research, assisted in data analysis, and preparation of the manuscript. All authors contributed to the article and approved the submitted version.

## Glossary

**Table:**

ABC	avidin-biotin complex
AK7	adenylate kinase 7
AMCF-II	alveolar macrophage chemotactic factor 2
APOA1	apolipoproteinA1
ANXA1	annexinA1
ATM	ataxia-telangiectasia-mutated
AUC	area under the curve
BGL	blood glucose levels
BMPR1B	bone morphogenetic protein receptor type 1B
CD	cluster of differentiation
CAV1	caveolin 1
CITED1	CREB-binding protein/p300-interacting transactivator with Asp/Glu-rich C-terminal domain 1
CK-7	cytokeratin-7
CNTN4	contactin 4
DAB	3, 3-diaminobenzidinetetrahydrochloride
ECM	extracellular matrix
EGTA	ethylene glycol-bis(&beta;-aminoethyl ether)-N,N,N&rsquo;,N&rsquo;-tetraacetic acid
GC	group-specific component
GO	gene ontology
HBSS	Hank&rsquo;s balanced salt solution
HEPES	4-(2-hydroxyethyl)-1-piperazineethanesulfonic acid
HP	horseradish peroxidase
I.E.	islet equivalent
i.p.	intraperitoneal
IL	interleukin
IPA	ingenuity pathway analysis
LFA-1	lymphocyte function-associated antigen 1
MMP	matrix metallopeptidase
NEK4	NIMA-related expressed kinase 4
NEUROD1	neurogenic differentiation 1
NIMA	never in mitosis gene A
OGTT	oral glucose tolerance test
PBMCs	peripheral blood mononuclear cells
PGHS-2	prostaglandin-endoperoxide H synthase 2
SWC9	swine workshop cluster 9
TGFB1	transforming growth factor beta 1
TLR4	toll-like receptor 4
VDBP	vitamin-D binding protein
VDCCs	voltage dependent calcium channels
