# Supplementary material for: Yield, cell composition, and function of islets isolated from different ages of neonatal pigs
Source: Front Endocrinol (Lausanne). 2022 Dec 21;13:1032906. doi: 10.3389/fendo.2022.1032906 (PMC9811407; doi:10.3389/fendo.2022.1032906)
Supplement: Supplementary file 1 [file Table_1.pdf]

Supplementary Table 1: Up-regulated genes in islets from 7-day-old pigs compared to islets from 3-day-old pigs

Gene Symbol	Corrected p-value	p-value	Fold change	Log Fold change	[3](normalized)	[7](normalized)	Entrez Gene	Probe Set ID
MMP3	0.003978	0.003612	9.13	3.19	-1.81	1.38	396769	Ssc.15927.2.S1_at
MMP3	0.003372	0.002907	8.22	3.04	-1.65	1.39	396769	Ssc.15927.1.S1_at
AMCF-II	0.000582	0.000086	7.73	2.95	-1.80	1.15	396900	Ssc.719.1.S1_at
MMP1	0.001319	0.000864	7.51	2.91	-1.74	1.17	397320	Ssc.16013.1.S1_at
PGHS-2	0.000723	0.000324	6.85	2.78	-1.20	1.57	397590	Ssc.7314.1.A1_at
AMCF-II	0.000582	0.000130	6.11	2.61	-1.49	1.12	396900	Ssc.719.1.S1_a_at
UFBP	0.000582	0.000061	6.01	2.59	-1.60	0.99	100170130	Ssc.12341.1.S1_at
F13A1	0.006939	0.006620	4.18	2.07	-0.69	1.37	100153504	Ssc.26345.1.S1_at
MMP3	0.000861	0.000465	4.08	2.03	-0.94	1.09	396769	Ssc.15927.2.A1_at
COL14A1	0.000534	0.000037	3.94	1.98	-1.07	0.91	100158059	Ssc.31124.1.S1_at
BLNK	0.000582	0.000119	3.85	1.94	-0.93	1.02	100152350	Ssc.8594.1.A1_at
NPY	0.000747	0.000353	3.69	1.88	-1.00	0.88	397304	Ssc.15981.1.S1_at
DCN	0.002048	0.001506	3.43	1.78	-1.10	0.68	396957	Ssc.10245.2.A1_a_at
PGHS-2	0.000222	0.000005	3.38	1.76	-0.83	0.93	397590	Ssc.23994.1.A1_at
NID2	0.000691	0.000271	3.35	1.74	-0.94	0.80	100156994	Ssc.1053.1.S1_at
SOX18	0.000582	0.000047	3.31	1.73	-0.89	0.84	100049667	Ssc.15360.1.A1_a_at
CDH5	0.000691	0.000285	3.28	1.72	-0.77	0.95	414737	Ssc.75.1.S1_at
STC1	0.000951	0.000557	3.19	1.67	-0.78	0.89	100125345	Ssc.2464.1.S1_at
SLPI	0.002033	0.001472	3.16	1.66	-0.93	0.73	396886	Ssc.6080.1.S1_at
FGL2	0.001160	0.000707	3.12	1.64	-0.70	0.94	448808	SscAffx.9.1.S1_at
THBD	0.000951	0.000557	3.11	1.64	-0.72	0.92	100157642	Ssc.20711.1.S1_at
APOD	0.004090	0.003761	3.11	1.64	-0.49	1.15	100157318	Ssc.5364.1.S1_at
RGS5	0.001213	0.000753	3.07	1.62	-0.71	0.91	397640	Ssc.8330.1.S1_at
AWN	0.000691	0.000302	3.07	1.62	-0.80	0.82	396783	Ssc.19852.1.S1_at
STMN2	0.000691	0.000292	3.03	1.60	-0.76	0.84	100156768	Ssc.13915.1.S1_at
AREG	0.008722	0.008722	3.01	1.59	-0.37	1.22	397668	Ssc.14467.2.S1_a_at
STMN2	0.002398	0.001847	2.98	1.58	-0.84	0.74	100156768	Ssc.3937.1.S1_at
CXCL14	0.003815	0.003377	2.98	1.57	-0.85	0.72	494467	Ssc.4984.1.S1_at
FGL2	0.002759	0.002223	2.89	1.53	-0.48	1.05	448808	Ssc.12579.1.A1_s_at
CAV1	0.000582	0.000134	2.86	1.51	-0.65	0.86	404693	Ssc.16989.1.A1_at
ITGB3	0.000684	0.000228	2.85	1.51	-0.84	0.66	397063	Ssc.44.1.S1_at

PECAM1	0.000222	0.000004	2.81	1.49	-0.78	0.71	396941	Ssc.6050.1.A1_at
CXCL2	0.000619	0.000192	2.77	1.47	-0.79	0.68	414904	Ssc.4871.1.S1_at
COL12A1	0.000944	0.000532	2.75	1.46	-0.81	0.65	100156689	Ssc.1049.1.S1_at
CXCL14	0.003166	0.002657	2.75	1.46	-0.73	0.72	494467	Ssc.20578.1.S1_at
IGFBP7	0.000747	0.000378	2.72	1.44	-0.67	0.77	100302573	Ssc.17186.2.S2_at
CCL2	0.006522	0.006147	2.70	1.43	-0.81	0.62	397422	Ssc.657.1.A1_at
ANXA1	0.001703	0.001209	2.69	1.43	-0.67	0.76	396942	Ssc.14559.1.S1_at
AXL	0.001098	0.000656	2.69	1.43	-0.72	0.71	100144875	Ssc.6566.1.A1_at
PBD-2	0.002219	0.001683	2.68	1.42	-0.69	0.73	404699	Ssc.21145.1.S1_at
RPS17	0.001362	0.000908	2.67	1.42	-0.77	0.65	414392	Ssc.942.1.S1_at
TBX3	0.000635	0.000204	2.66	1.41	-0.69	0.72	100152741	Ssc.6578.1.S1_at
KNG1	0.000817	0.000432	2.65	1.41	-0.73	0.68	396568	Ssc.24401.1.A1_s_at
CD93 /// LOC100152403	0.000691	0.000269	2.65	1.41	-0.64	0.76	100152403 /// 100155660	Ssc.4681.1.S1_at
PTGS1	0.000691	0.000263	2.60	1.38	-0.56	0.82	397541	Ssc.1986.1.S1_at
ICA	0.001476	0.001018	2.59	1.37	-0.81	0.56	396845	Ssc.16562.1.S1_at
AKAP12	0.000587	0.000169	2.57	1.36	-0.73	0.63	100152595	Ssc.7802.1.A1_at
CLDN1	0.000587	0.000147	2.54	1.35	-0.55	0.79	396566	Ssc.9467.1.S1_at
COL14A1	0.002509	0.001990	2.48	1.31	-0.75	0.56	100158059	Ssc.15374.1.S1_at
IGFBP7	0.000817	0.000429	2.47	1.31	-0.62	0.68	100302573	Ssc.17186.2.S1_at
LAMA4	0.000534	0.000037	2.46	1.30	-0.71	0.59	100154581	Ssc.24909.1.S1_at
ST8SIA4	0.000582	0.000106	2.44	1.28	-0.68	0.60	641356	Ssc.5677.1.A1_at
IFI16	0.000691	0.000268	2.44	1.28	-0.48	0.80	100156073	Ssc.10884.1.A1_at
MMP-2	0.002064	0.001542	2.43	1.28	-0.63	0.65	397391	Ssc.5713.1.S1_at
CAV2	0.000747	0.000373	2.40	1.27	-0.50	0.77	100125375	Ssc.31165.1.S1_at
MATN2	0.003761	0.003285	2.39	1.26	-0.64	0.62	100155607	Ssc.7756.1.A1_at
SPARCL1	0.000582	0.000126	2.37	1.25	-0.68	0.57	100037275	Ssc.6531.1.A1_at
LAMA2	0.000582	0.000090	2.37	1.25	-0.57	0.68	100154420	Ssc.11623.1.A1_at
STC1	0.003339	0.002840	2.36	1.24	-0.65	0.59	100125345	Ssc.15105.1.S1_at
LIF	0.000691	0.000296	2.34	1.22	-0.66	0.56	399503	Ssc.21256.1.S2_at
ANGPTL2	0.001319	0.000844	2.33	1.22	-0.64	0.58	100126164	Ssc.29929.1.S1_at
IL8	0.007690	0.007442	2.32	1.21	-0.68	0.54	396880	Ssc.658.1.S1_at
TGFB1	0.001319	0.000856	2.32	1.21	-0.55	0.66	397078	Ssc.76.3.S1_a_at
CXCL2	0.001703	0.001213	2.32	1.21	-0.60	0.61	396594	Ssc.19692.1.S1_at
CAV2	0.000747	0.000355	2.32	1.21	-0.47	0.75	100125375	Ssc.1291.1.A1_at

ASS1	0.005979	0.005567	2.30	1.20	-0.54	0.67	414411	Ssc.17717.1.S1_at
CPB1	0.001476	0.001010	2.30	1.20	-0.52	0.68	397341	Ssc.16048.1.S1_at
CDH5	0.000587	0.000164	2.28	1.19	-0.46	0.73	414737	Ssc.75.2.S1_a_at
LAMA2	0.000944	0.000521	2.22	1.15	-0.63	0.53	100154420	Ssc.11815.1.A1_s_at
MYLK	0.000582	0.000121	2.20	1.14	-0.59	0.55	100152091	Ssc.23247.1.S1_at
ICAM-1	0.000582	0.000098	2.19	1.13	-0.52	0.61	396750	Ssc.8997.1.A1_at
C1R	0.000619	0.000188	2.18	1.12	-0.53	0.60	445464	Ssc.983.1.S1_at
GRK5	0.000587	0.000159	2.17	1.12	-0.60	0.51	100152937	Ssc.30072.1.A1_at
IL1A	0.003867	0.003467	2.15	1.10	-0.42	0.69	397094	Ssc.113.1.S1_at
ICAM-1	0.000582	0.000100	2.14	1.09	-0.42	0.67	396750	Ssc.11187.1.S1_at
TMEFF2	0.002759	0.002252	2.12	1.09	-0.50	0.59	654831	Ssc.19235.1.A1_at
TLR4	0.000582	0.000105	2.11	1.08	-0.56	0.52	399541	Ssc.12781.1.A2_at
APOA1	0.000691	0.000266	2.09	1.06	-0.58	0.48	397691	Ssc.807.1.S1_at
CAV1	0.002412	0.001885	2.08	1.06	-0.40	0.66	404693	Ssc.12842.1.S1_at
HSD11B1	0.000587	0.000165	2.08	1.05	-0.66	0.39	397480	Ssc.777.1.S1_at
BACE2	0.000534	0.000027	2.07	1.05	-0.54	0.51	100517374	Ssc.10143.1.A1_at
PLET	0.007690	0.007513	2.07	1.05	-0.57	0.48	396570	Ssc.2506.1.S1_at
BMPR1B	0.003156	0.002612	2.07	1.05	-0.59	0.46	396691	Ssc.66.1.S3_at
BACE2	0.000747	0.000376	2.07	1.05	-0.61	0.43	100517374	Ssc.18206.1.S1_at
PECAM1	0.000534	0.000019	2.06	1.04	-0.49	0.56	396941	Ssc.14558.1.S1_at
STEAP1	0.008627	0.008528	2.03	1.02	-0.48	0.54	397573	Ssc.1183.1.S1_at
DSE	0.000582	0.000117	2.02	1.01	-0.54	0.47	100520877	Ssc.26182.1.S1_at
