# Supplementary material for: Yield, cell composition, and function of islets isolated from different ages of neonatal pigs
Source: Front Endocrinol (Lausanne). 2022 Dec 21;13:1032906. doi: 10.3389/fendo.2022.1032906 (PMC9811407; doi:10.3389/fendo.2022.1032906)
Supplement: Supplementary file 2 [file Table_2.pdf]

Supplementary Table 2: Down-regulated genes in islets from 7-day-old pigs compared to islets from 3-day-old pigs

Gene Symbol	Corrected p-value	p-value	Fold change	Log Fold change	[3](normalized)	[7](normalized)	Entrez Gene	Probe Set ID
AK7	0.000232	0.000086	-2.87	-1.52	0.83	-0.69	100152573	Ssc.12884.1.A1_at
GC	0.000679	0.000291	-2.65	-1.40	0.59	-0.82	448964	Ssc.2992.1.A1_at
CITED1	0.000039	0.000006	-2.60	-1.38	0.67	-0.71	654297	Ssc.3326.1.S1_at
NEK4	0.000017	0.000002	-2.51	-1.33	0.70	-0.63	100525954	Ssc.5624.1.A1_at
CNTN4	0.000099	0.000021	-2.50	-1.32	0.71	-0.62	100153462	Ssc.6864.1.A1_at
NEUROD1	0.000127	0.000036	-2.41	-1.27	0.63	-0.64	397283	Ssc.15925.1.S1_at
ZNF280D	0.001552	0.001108	-2.38	-1.25	0.63	-0.62	100155694	Ssc.30327.1.A1_at
LRRC49	0.000807	0.000500	-2.37	-1.24	0.75	-0.50	100154470	Ssc.13335.1.A1_at
FXYD3	0.001574	0.001199	-2.34	-1.22	0.52	-0.70	397413	Ssc.569.1.S1_at
FBXO5	0.000017	0.000001	-2.27	-1.18	0.64	-0.55	100514379	Ssc.24858.1.S1_at
ASCL2	0.000232	0.000088	-2.26	-1.18	0.63	-0.55	100144467	Ssc.19166.1.S1_s_at
GALP	0.009205	0.008767	-2.20	-1.14	0.46	-0.68	396772	Ssc.4875.1.S1_at
HOPX	0.001267	0.000845	-2.17	-1.12	0.64	-0.48	396692	Ssc.383.1.S1_at
CDO1	0.000714	0.000353	-2.16	-1.11	0.65	-0.47	100312964	Ssc.7106.1.S1_at
ADRA2A	0.003586	0.003073	-2.16	-1.11	0.44	-0.67	399501	Ssc.27594.1.S1_at
ME1	0.003932	0.003558	-2.13	-1.09	0.61	-0.48	397538	Ssc.16336.1.S2_at
CACNA2D1	0.000714	0.000381	-2.11	-1.07	0.58	-0.49	397377	Ssc.16114.1.S1_at
ZNF133	0.000099	0.000024	-2.10	-1.07	0.54	-0.53	100523338	Ssc.27583.1.S1_at
TTR	0.009299	0.009299	-2.06	-1.04	0.20	-0.84	397419	Ssc.640.1.S1_at
KIF11	0.000714	0.000408	-2.06	-1.04	0.56	-0.49	100526080	Ssc.7361.1.A1_at
PC2	0.002924	0.002367	-2.01	-1.01	0.28	-0.72	445533	Ssc.109.1.S1_at
