# Supplementary material for: Yield, cell composition, and function of islets isolated from different ages of neonatal pigs
Source: Front Endocrinol (Lausanne). 2022 Dec 21;13:1032906. doi: 10.3389/fendo.2022.1032906 (PMC9811407; doi:10.3389/fendo.2022.1032906)
Supplement: Supplementary file 3 [file DataSheet_1.pdf]

Supplementary Material

1 Primary and secondary antibodies used for immunostaining of dissociated islets and formalin-fixed paraffin-embedded samples

Primary antibody	Dilution	Vendor
Guinea Pig anti-pig insulin	1:1000	DAKO Laboratories, Mississauga, ON, Canada
Guinea pig anti-pig glucagon	1:5000	Linco Research, Inc., St. Charles, Missouri, USA
Goat anti-human somatostatin	1:1000	Santa Cruz Biotechnology Inc., Santa Cruz, California, USA
Rabbit anti-human pancreatic polypeptide	1:1000	DAKO Laboratories
mouse anti-human CK-7	1:200	DAKO Laboratories

Secondary antibody	Dilution	Vendor
Goat Anti-Guinea Pig IgG Antibody (H+L), Biotinylated	1:200	Vector Laboratories, Burlingame, CA, USA
Horse Anti-Goat IgG Antibody (H+L), Biotinylated	1:200	Vector Laboratories, Burlingame, CA, USA
Goat Anti-Rabbit IgG Antibody (H+L), Biotinylated	1:200	Vector Laboratories, Burlingame, CA, USA
Goat Anti-Mouse IgG Antibody (H+L), Biotinylated	1:200	Vector Laboratories, Burlingame, CA, USA

2 Recipe of solutions used for measurement of Kv channel and voltage-dependent K^+ and Ca^{2+} channel activities

Salts (mmol/l)	Kv intracellular solution	Kv extracellular solution	VDCC intracellular solution	VDCC extracellular solution
NaCl (mmol/l)	---	135	---	100
KCl (mmol/l)	140	5.4	---	---
$CaCl_2$ (mmol/l)	1	1	---	---
$MgCl_2$ (mmol/l)	1	1.2	1	1
$BaCl_2$ (mmol/l)	---	---	---	20
CsCl (mmol/l)	---	---	---	5
HEPES (mmol/l)	10	10	20	10
EGTA (mmol/l)	10	---	5	---
ATP-Mg (mmol/l)	3	---	3	---
Glucose (mmol/l)	---	5	---	5
Cs glutamate (mmol/l)	---	---	140	---
tetraethyl ammonium chloride (mmol/l)	---	---	20	---
Tetrodotoxin (µmol/l)	---	---	---	0.5
pH	7.3 (with KOH)	7.3 (with NaOH)	7.3 (with CsOH)	7.35 (with CsOH).
