# Supplementary material for: Yield, cell composition, and function of islets isolated from different ages of neonatal pigs
Source: Front Endocrinol (Lausanne). 2022 Dec 21;13:1032906. doi: 10.3389/fendo.2022.1032906 (PMC9811407; doi:10.3389/fendo.2022.1032906)
Supplement: Supplementary file 4 [file DataSheet_2.pdf]

Supplementary Figure 1

Figure 1:

A

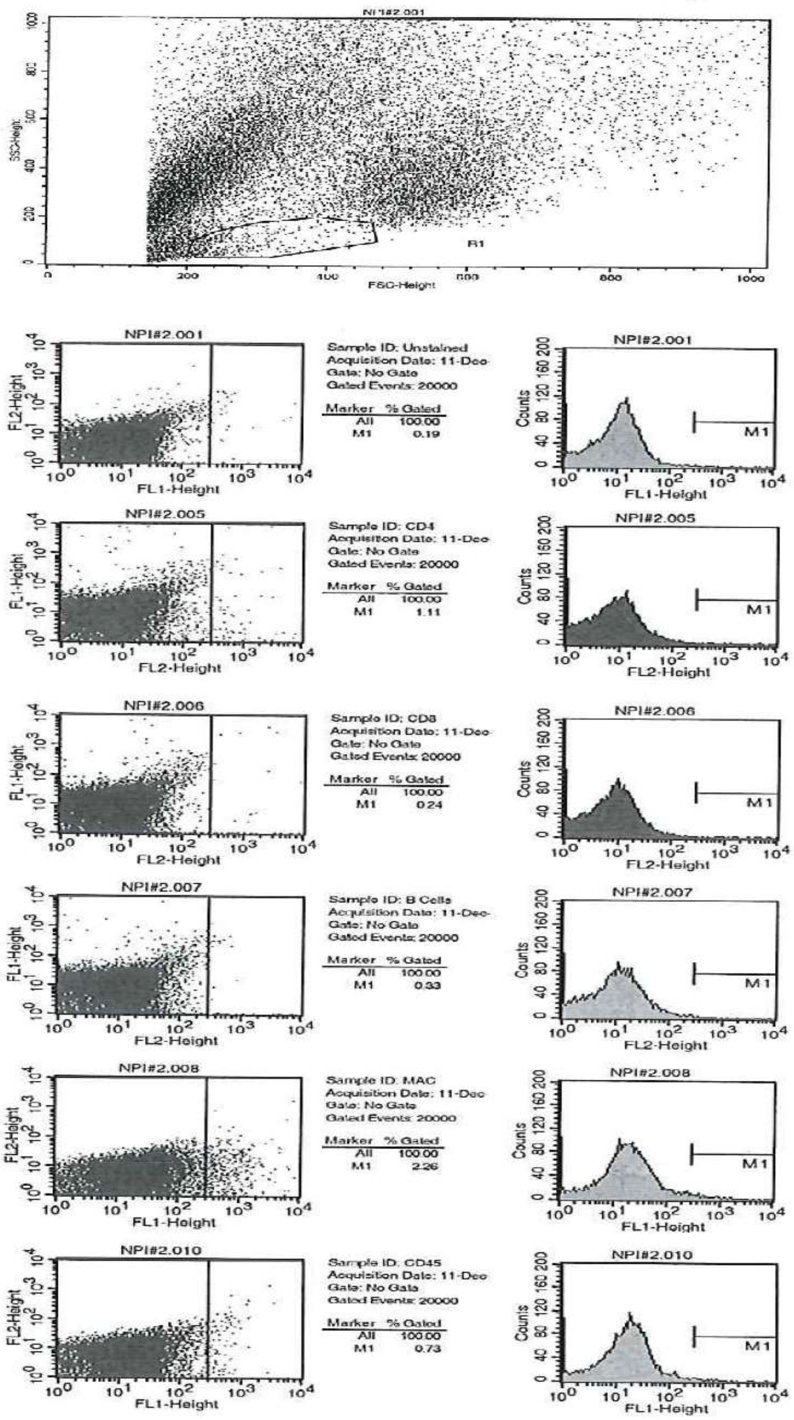

B

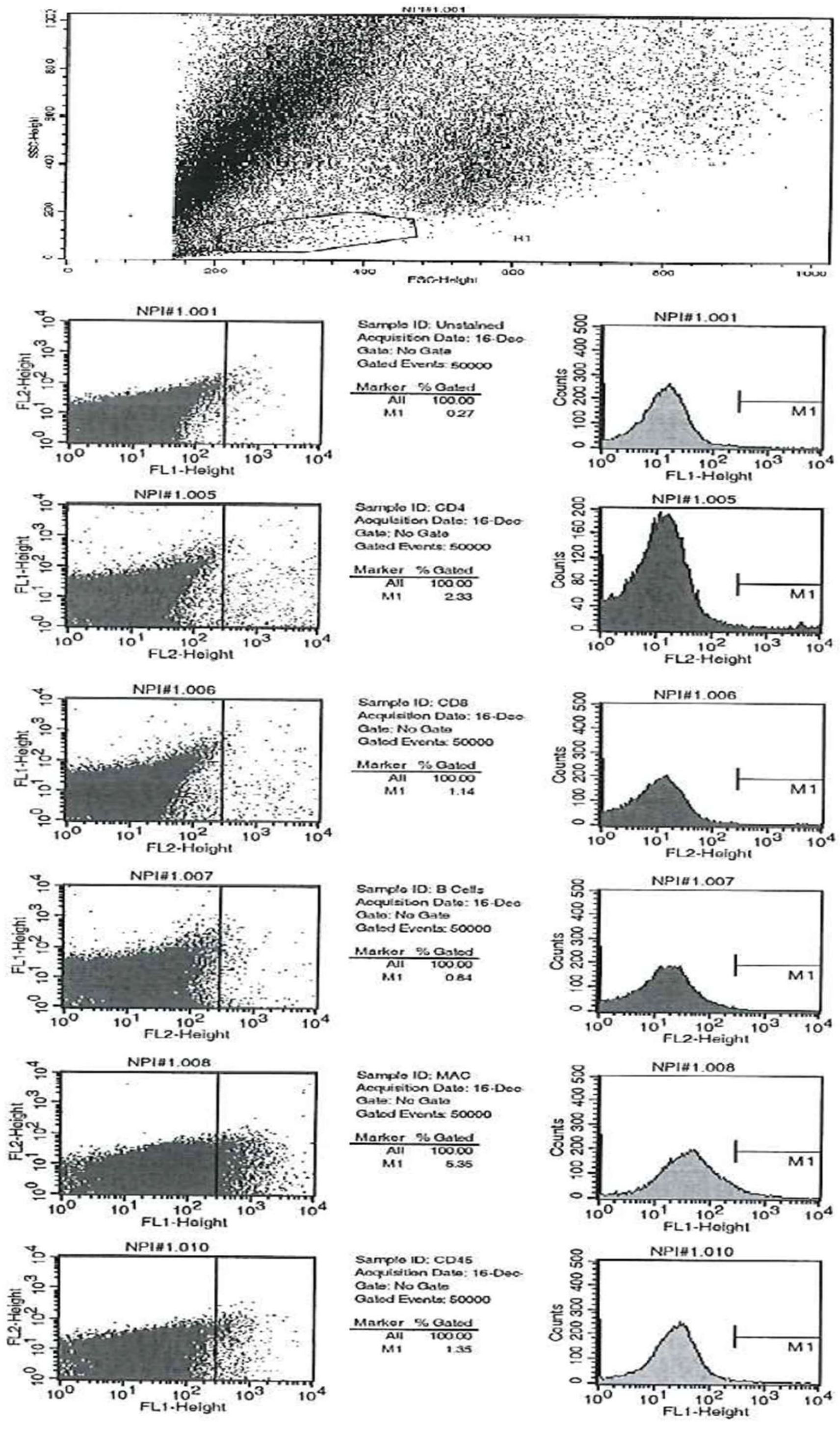
SSC-Height
FSC-Height
R1
NPI#1.001
FL1-Height
FL2-Height
Counts
M1
Sample ID: Unstained
Acquisition Date: 16-Dec
Gate: No Gate
Gated Events: 50000
Marker % Gated
All 100.00
M1 0.27
NPI#1.005
Sample ID: CD4
Acquisition Date: 16-Dec
Gate: No Gate
Gated Events: 50000
Marker % Gated
All 100.00
M1 2.33
NPI#1.006
Sample ID: CD8
Acquisition Date: 16-Dec
Gate: No Gate
Gated Events: 50000
Marker % Gated
All 100.00
M1 1.14
NPI#1.007
Sample ID: B Cells
Acquisition Date: 16-Dec
Gate: No Gate
Gated Events: 50000
Marker % Gated
All 100.00
M1 0.84
NPI#1.008
Sample ID: MAC
Acquisition Date: 16-Dec
Gate: No Gate
Gated Events: 50000
Marker % Gated
All 100.00
M1 5.35
NPI#1.010
Sample ID: CD45
Acquisition Date: 16-Dec
Gate: No Gate
Gated Events: 50000
Marker % Gated
All 100.00
M1 1.35

C

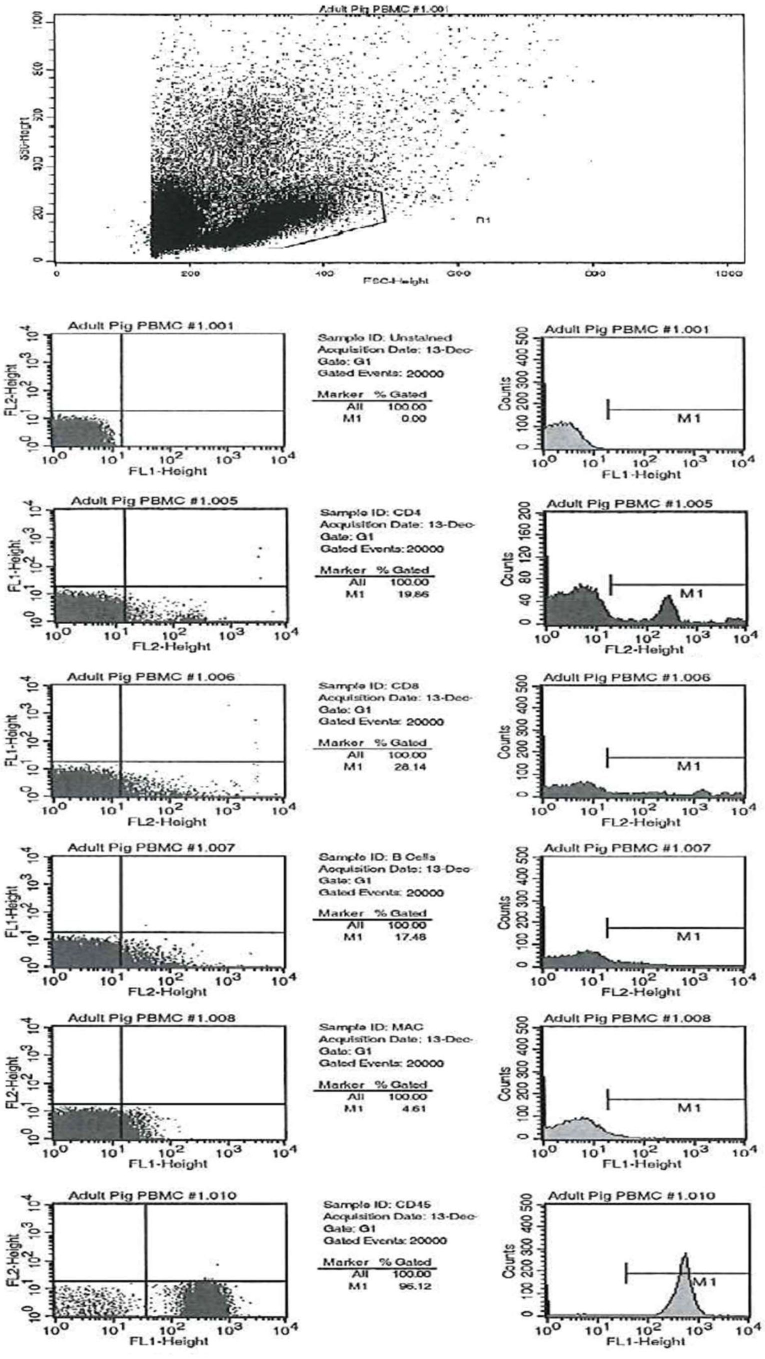

Supplementary Figure 1. Representative flow cytometry scatter plots and histograms. Data were acquired by running the samples on the BD FACS Calibur machine (BD Biosciences, Ontario, Canada) flow cytometer with 20,000, 50,000, and 20,000 events collected for 3-day pig islets (A), 7-day pig islets (B), and adult PBMCs (C), respectively, in which the cells were first gated using the forward scatter (FSC) and side scatter (SSC) to find viable single-cell events. The data were analyzed using the BD FACSDivaTM software (BD Biosciences, Mississauga, Ontario). The events were

further analyzed for the expression of markers by fluorescence and the percent of each cell population queried was automatically generated by the software in each quadrant. Untreated served as negative controls. Adult pig PBMCs were used as a positive control. The antibodies which were used in this assay are listed below:

Antibody	Dilution	Vendor
Mouse Anti-Porcine CD4-PE conjugated	1:100	Southern Biotech, Birmingham, AL, USA
Mouse Anti-Porcine CD8α-PE conjugated	1:100	Southern Biotech, Birmingham, AL, USA
Mouse anti-B Cell (sIg) antibody, PE conjugated	1:100	Abcam Inc., Cambridge, MA, USA
Mouse anti-pig SWC9 (CD203a) antibody, FITC conjugated	1:100	Serotec Ltd., Kidlington, OX, UK
Mouse anti-pig CD45 antibody, FITC conjugated	1:100	Antigenix America, Inc., Huntington Station, NY, USA
