# Supplementary material for: Yield, cell composition, and function of islets isolated from different ages of neonatal pigs
Source: Front Endocrinol (Lausanne). 2022 Dec 21;13:1032906. doi: 10.3389/fendo.2022.1032906 (PMC9811407; doi:10.3389/fendo.2022.1032906)
Supplement: Supplementary file 5 [file DataSheet_3.pdf]

Supplementary Figure 2

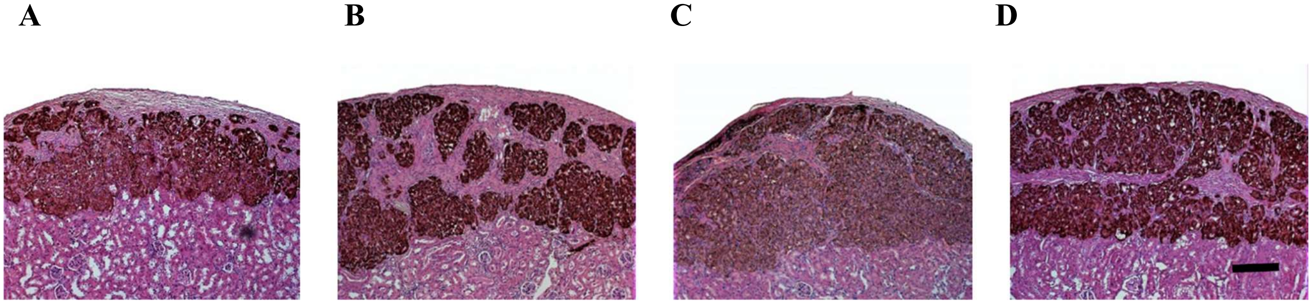

Supplementary Figure 2. Representative islet graft from B6 *rag-/-* mice transplanted with 2000 I.E. islets from 3-, 5-, 7-, and 10-day-old pigs at >150 days post-transplantation. Brown-stained structures represent intact islets with insulin-positive beta cells. Scale bar represents 100 µm.
